# Organo-catalyzed deamination of polystyrene sulfonamide for diverse post-polymerization modification of styrenic polymers

**DOI:** 10.1039/d5py01024a

**Published:** 2026-04-01

**Authors:** Tulaja Shrestha, Dan My Nguyen, Harrison Goehrig, Vidhika S. Damani, Caitlin M. Quinn, Laure V. Kayser

**Affiliations:** a Department of Chemistry and Biochemistry, University of Delaware Newark DE 19711 USA lkayser@udel.edu; b Department of Materials Science and Engineering, University of Delaware Newark DE 19711 USA

## Abstract

Post-polymerization modification allows for the incorporation of functional groups that would otherwise be incompatible with polymerization conditions, enhancing synthetic efficiency and facilitating the creation of complex polymer architectures for specialized applications, such as biomedical devices, electronics, and advanced coatings. Herein, we report a method inspired by late-stage functionalization of small molecules for the post-polymerization modification of aromatic polymers, specifically polystyrene (PS), under mild reaction conditions. First, PS was converted to polystyrene sulfonamide (PSSNH_2_) with an 85% yield using established procedures. PSSNH_2_ was subsequently transformed into a reactive sulfinate by deamination using an N-heterocyclic carbene (NHC) catalyst and benzaldehyde. The catalytic process was optimized by varying catalysts, solvents, bases, temperatures, and reaction times. The highest degree of deamination was 88% with a bicyclic NHC and K_2_CO_3_ base in DMSO for 18 hours at 120 °C. The reactive sulfinate was then treated with various functional reagents, resulting in a library of aromatic polymers with different substituents with high degrees of functionalization ranging from 74% to 98%. Similarly, we modified expanded PS waste with trifluorobutyl iodide with a degree of functionalization of 72%, highlighting a new avenue for plastic upgrading. This approach could be used to rapidly generate functionalized polymers from PS with potential applications, including antibacterial properties and flame retardancy.

## Abstract 2

Post-polymerization modification of polystyrene generates sulfinate intermediates that enable modular diversification to a wide range of functional groups.

## Graphical abstract

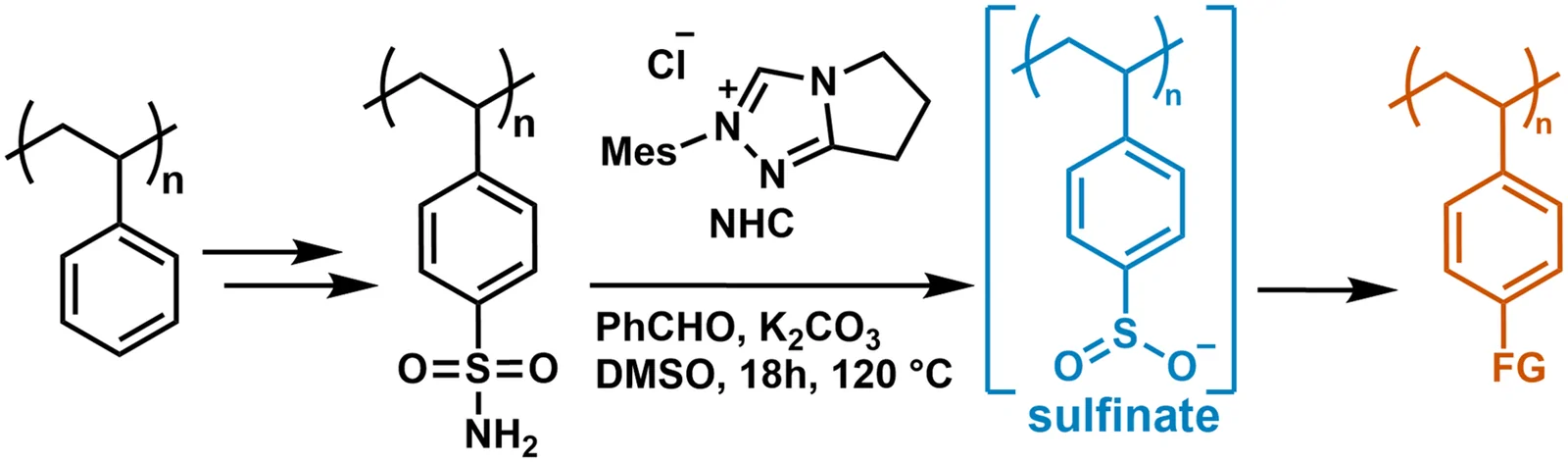

## Introduction

Post-polymerization modification (PPM) is crucial in polymer chemistry because it allows for the precise tailoring of polymer properties after the initial polymerization process.^1–4^ This flexibility is essential for incorporating functional groups incompatible with the polymerization conditions, thereby expanding the range of possible polymer functionalities. It also enhances the efficiency of synthetic processes, enabling the introduction of desired functionalities.^5^ PPMs employ efficient organic synthetic strategies such as transition metal catalysis,^6,7^ click chemistry,^8–10^ photoredox catalysts,^3^ nucleophilic aromatic substitutions,^11–15^ and radical reactions.^16^ These innovations have enabled the creation of complex polymer architectures and high-value materials with specialized applications, such as biomedical devices,^1,2^ electronics,^3^ and advanced coatings.^4^ By enabling sequential modifications, PPM also allows for the stepwise introduction of multiple functional groups, leading to polymers with specific and enhanced properties.

Over the past two decades, PPM has also matured into a methodology for functionalizing specialty polymers, including bio-derived materials,^17^ polypeptides,^18^ and synthetic scaffolds with thiol,^19^ ester, or halide handles.^20^ Such systems have enabled tunable properties in biomaterials and therapeutics, high-throughput functional libraries *via* amidation or thiol–ene reactions, and solid-state modifications of engineering thermoplastics without compromising processability.^21–23^ These developments illustrate the broad reach of PPM across diverse classes of polymers. PPM has also opened new avenues for repurposing commodity plastics.^24–26^ For example, Golder and co-workers demonstrated the functionalization of polybutadiene to its thermal and surface-wetting properties.^27^ Vlachos and co-workers reported a plasma-based oxidation approach for polyethylene upcycling.^28^ Watson and co-workers fluorinated poly(acrylic acid) in water under organophotoredox conditions using vitamin B_2_ as a green catalyst.^29^

Polystyrene (PS) is produced on a global scale exceeding 20 million metric tons annually, therefore representing an attractive target for PPM. Numerous strategies have been pursued to functionalize PS. Early approaches, such as Friedel–Crafts acylation^13^ and benzylic azidation with hypervalent iodine reagents,^30^ achieved limited conversions and often compromised molecular weight. More recently, Leibfarth and coworkers reported a photocatalytic fluoroalkylation of PS, featuring *in situ* generation of perfluoroalkyl species, followed by Ru(bpy)Cl_2_-catalyzed functionalization that afforded up to 72% conversion while preserving the polymer's molecular weight.^31^ Additionally, a study conducted by Xue *et al.* demonstrated a mild arylation strategy with a high degree of functionalization, approaching nearly 100% conversion.^5^ Chan and co-workers reported a two-step amination strategy to access antibacterial PS coatings,^32^ while Liu *et al.* introduced phosphorylation to tune surface and thermal properties, introducing flame-retardant properties in polystyrene.^33^ We previously developed a sulfonation method which preserved the integrity of the parent PS. The resulting polystyrene sulfonate (PSS) was subsequently complexed with a conjugated polymer and used in organic electrochemical transistors and silicon-based hybrid solar cells.^34^ These examples highlight the progress toward milder, more versatile transformations. While most of these methods have limited functional group scope, a recent study by Wang and co-workers reported the bromination of commercial-grade polystyrene under mild reaction conditions using AuCl_3_ as a catalyst, thereby preserving the polymeric backbone. This brominated polymer served as a lynchpin precursor that could be diversified into a range of functionalized derivatives. Such strategies underscore the value of lynchpin intermediates where a single reactive functionality enables broad diversification under mild conditions.^35^ Towards this goal, most studies rely on transition-metal catalysts which may be complex to remove from the final polymer motivating the development of complementary, metal-free methods.

**Scheme 1:**
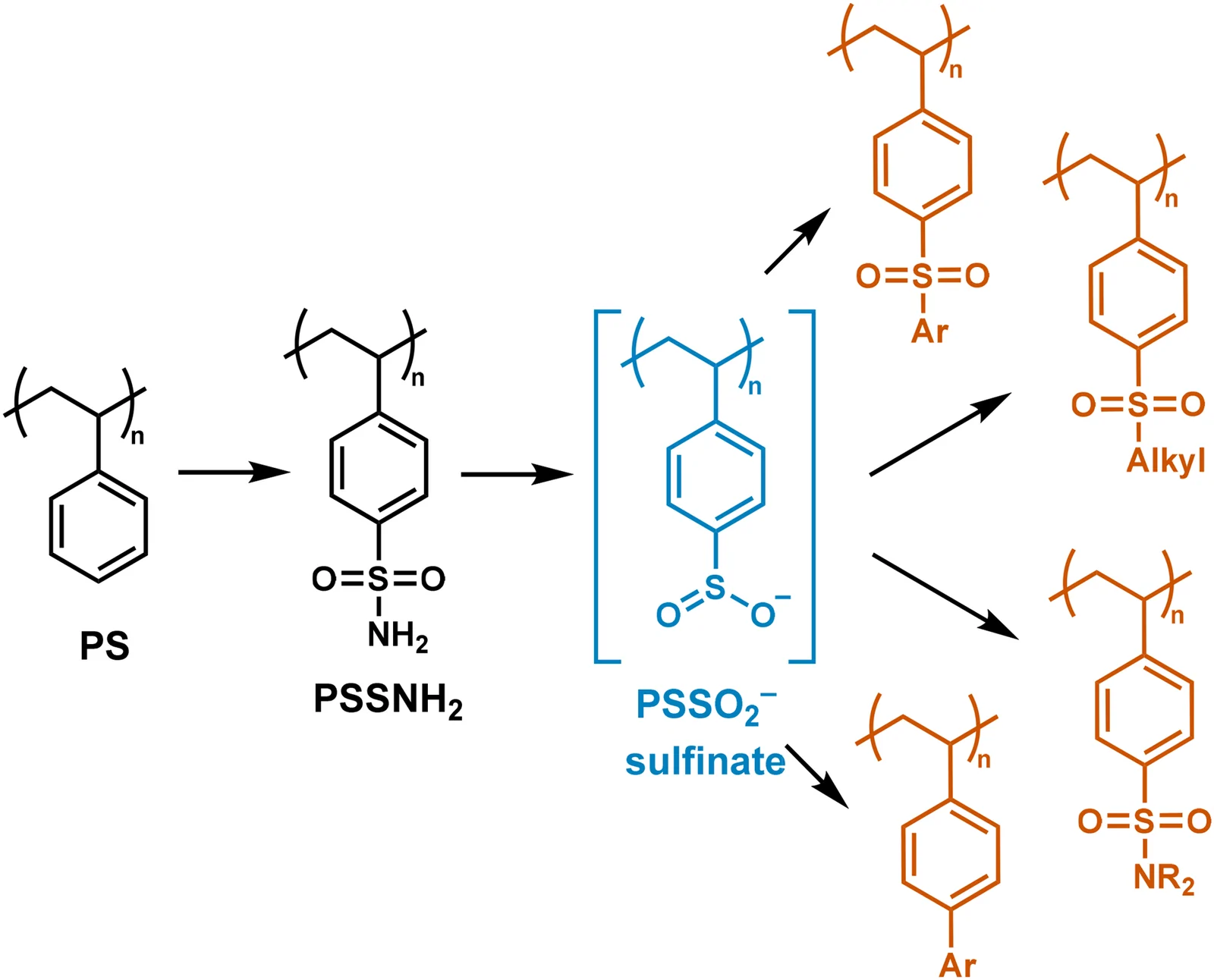
Post-polymerization modification (PPM) of polystyrene by installation of a reactive sulfinate group to access a diverse library of polymers.

Here, we present an organocatalytic PPM strategy that installs polystyrene sulfinate (PSSO_2_^−^) as a lynchpin *via* N-heterocyclic carbene (NHC)-catalyzed deamination of PS sulfonamides (Scheme 1). Adapted from a small molecule synthesis originally developed by Fier *et al.* for small molecules, this approach generates sulfinates under mild conditions without the use of transition metals.^36^ The sulfinate intermediate engages with diverse electrophiles and participates in cross-coupling reactions, giving rise to alkyl and aryl sulfones, sulfonamides, and biaryls. By consolidating multiple functionalization pathways into a single precursor, this lynchpin-based, organocatalytic strategy expands the synthetic toolbox for PS modification while complementing existing metal-catalyzed approaches. The breadth of transformations accessible from this intermediate could enable the preparation of polymers for advanced applications in antifouling coatings,^37,38^ separation membranes,^39^ biosensors,^40^ and flame-retardant materials.^41^

## Results and discussion

**Fig. 1:**
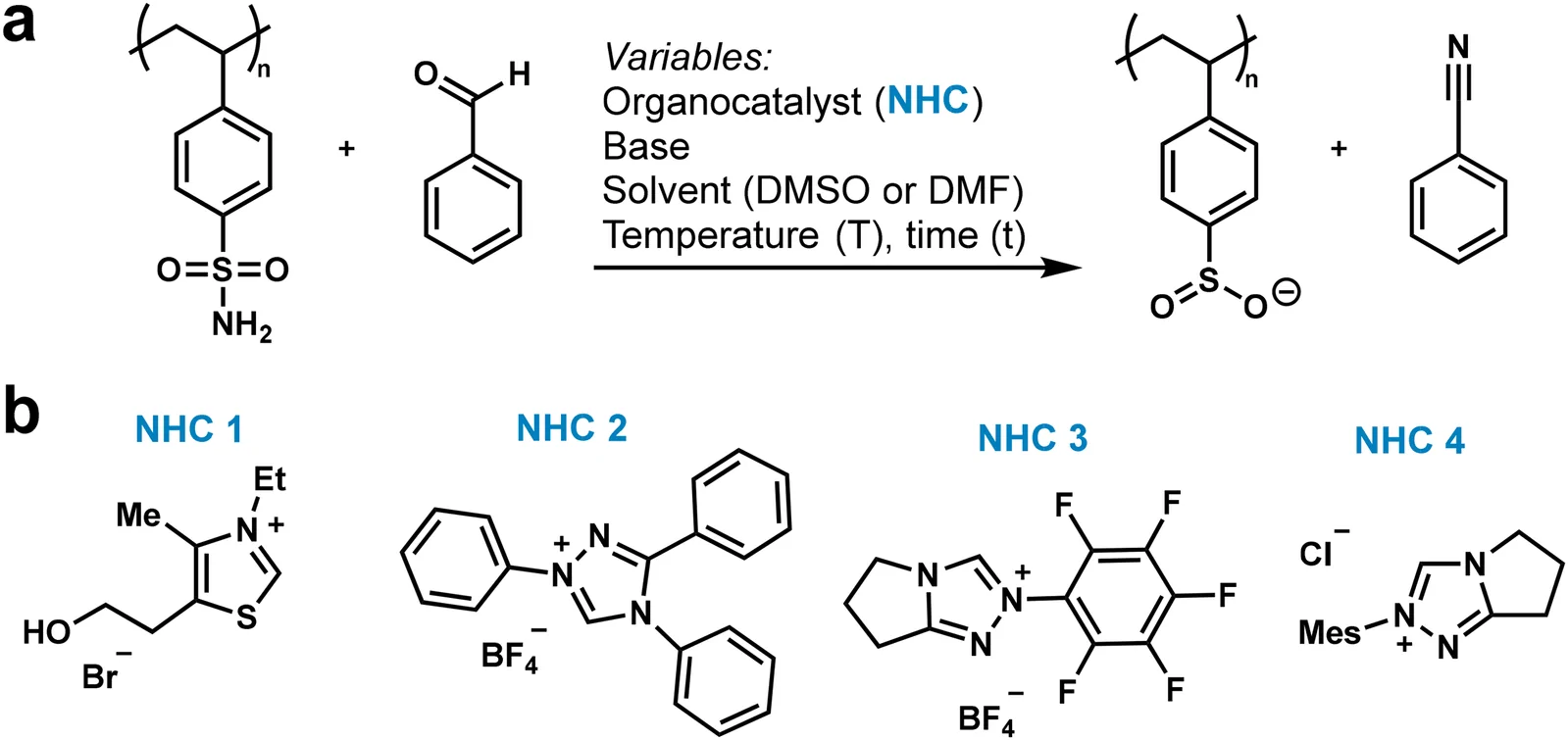
Optimization of the NHC-catalyzed deamination of polystyrene sulfonamide to obtain the reactive sulfinate intermediate. (a) Reaction scheme and variables. (b) Structures of the four NHCs. NHC 1: 3-ethyl-5-(2-hydroxyethyl)-4-methyl-1,3-thiazolium bromide, NHC 2: 1,4-*N*,*N*-diphenyl-3-phenyl triazol-1-ium tetrafluoroborate, NHC 3: 2-pentafluorophenyl-6,7-dihydro-5*H*-pyrrolo[2,1-*c*][1,2,4]triazol-2-ium tetrafluoroborate, NHC 4: 2-mesityl-2,5,6,7-tetrahydropyrrolo [2,1-*c*] [1,2,4]triazol-4-ium chloride.

To optimize the PPM, we focused on a model PS made by anionic polymerization with *M*_n_ = 19 kg mol^−1^ and low dispersity (*Đ* = 1.42), as measured by gel permeation chromatography (GPC) (Fig. S1), to ensure good solubility. The degree of polymerization (DP) was calculated to be 183. The PS was transformed to polystyrene sulfonamide (PSSNH_2_) using a commonly used sequential chlorosulfonation and amidation reactions (Scheme 1).^42^ PSSNH_2_ thus synthesized had a degree of sulfonation of >95% and degree of amidation of 85–92%, as determined by X-ray photoelectron spectroscopy (XPS). XPS and IR spectroscopy (Fig. S2) were used for characterization because PSSNH_2_ was insoluble in common solvents, such as toluene, trichlorobenzene, DCM, chloroform, DMF, DMSO, DMAc, pyridine, methanol, and ethyl acetate. The low solubility of the polymers prevented us from obtaining GPC data. As such, we cannot confirm that the polymer main chain did not undergo chain scission (reduction in molecular weight) or crosslinking (increase in molecular weight). In contrast to the parent polystyrene, the functionalized polymers exhibited very limited solubility in common organic solvents and only swelled in highly polar aprotic solvents such as DMSO and DMF. This behavior is likely influenced by the high density of polar functional groups introduced during post-polymerization modification, which can promote strong interchain interactions and aggregation. In addition, the incorporation of functional groups may increase chain rigidity and cohesive energy density, further suppressing molecular dissolution. While chemical crosslinking cannot be ruled out due to the absence of GPC data, the consistent observation of swelling rather than true dissolution suggests that physical association and morphological effects play a major role. XPS was chosen over elemental analysis for quantitative assessment of the degree of functionalization because it also provided a qualitative verification of the purity of the functionalized polymer (*i.e.*, difference in binding energy in reactant *versus* functionalized polymer). Following the successful synthesis and characterization of PSSNH_2_, we proceeded to optimize the NHC-catalyzed deamination of PSSNH_2_ (Fig. 1a and Table 1) to form polystyrene sulfinate (PSSO_2_^−^). This reaction proceeds through the attack of the nitrogen of the sulfonamide with the aldehyde to form an *N*-sulfonylimine intermediate, ultimately liberating a nitrile through decomposition as the stoichiometric byproduct along with the sulfinate product.^36^ Following the general reaction shown in Fig. 1a of PSSNH_2_ with benzaldehyde, we varied the NHC catalyst (NHC 1 to NHC 4, Fig. 1b), solvent, base, temperature, and time to find the reaction conditions with the highest conversion of sulfonamide to sulfinate. The extent of deamination was calculated by XPS by measuring the nitrogen : sulfur ratio in the polymers purified using Soxhlet washes in chloroform, methanol, and water for 18 hours each (Table 1). The purity of the PSSNH_2_ for the optimized reaction conditions of the deamination step was verified qualitatively using solid-state ^13^C and ^15^N NMR to ensure the successful purification steps. The presence of sharp peaks would indicate the presence of a small molecule and, hence, residual impurities. We did not observe such impurity peaks in the ^13^C-NMR (Fig. S3) nor the ^15^N-NMR (Fig. S4).

**Table 1:** Optimization of the reaction conditions for the NHC-catalyzed deamination

Entry	Solvent	NHC	Base	Time (h)	Temperature (°C)	% Deamination^a^	Yield (%)
1	DMSO	NHC 1	K_2_CO_3_	24	100	5	72
2	DMSO	NHC 2	K_2_CO_3_	24	100	39	79
3	DMSO	NHC 3	K_2_CO_3_	24	100	4	74
4	DMSO	NHC 4	K_2_CO_3_	24	100	86	85
5	**DMF**	NHC 4	K_2_CO_3_	24	100	47	81
6	DMSO	NHC 4	**Cs**_**2**_ **CO**_**3**_	24	100	30	75
7	DMSO	NHC 4	**NEt**_**3**_	24	100	38	82
8	DMSO	NHC 4	K_2_CO_3_	**12**	100	55	93
9	DMSO	NHC 4	K_2_CO_3_	**18**	100	67	91
10	DMSO	NHC 4	K_2_CO_3_	**48**	100	88	83
11	DMSO	NHC 4	K_2_CO_3_	24	**60**	33	74
12	DMSO	NHC 4	K_2_CO_3_	24	**80**	86	78
13	DMSO	NHC 4	K_2_CO_3_	24	**120**	88	79
14	DMSO	NHC 4	K_2_CO_3_	**12**	**120**	81	77
15	DMSO	NHC 4	K_2_CO_3_	**18**	**120**	86	85

a Calculated from XPS.

Four NHCs were chosen for this optimization study (entries 1–4) based on their documented performance in related transformations (Fig. 1a). NHC 1 was chosen due to its reported effectiveness in facilitating Stetter-type reactions where the NHC enables aldimine umpolung, making the aldimine nucleophilic. NHC 1 was found to work better with substrates involving arylated substrates.^43^ NHCs 2 and 3 were selected from the work of Sun *et al.*, where they achieved high yields (97% and 90%, respectively) in the reaction with tosylated aldimines to generate nitriles by desulfonating the imine. This mechanistic pathway is analogous to that proposed in our study.^44^ Although NHC 4 gave a comparatively lower yield (84%) in this article, it gave the highest yield in a separate report by Fier *et al.* on the deamination of sulfonamides.^36^ We therefore hypothesized that these four NHCs could lead to effective deamination of PSSNH_2_. NHC 1 (entry 1), NHC 2 (entry 2), NHC 3 (entry 3), and NHC 4 (entry 4) exhibited degrees of deamination of 5%, 39%, 4%, and 86% respectively, as determined using XPS. Similar to past reports with small molecules,^36^ the best NHC for the deamination was found to be NHC 4 (entry 4) with a significantly higher degree of deamination (86%) when the reaction was run at 100 °C for 24 hours in DMSO as the solvent and with K_2_CO_3_ as the base.

Next, we investigated the role of the solvent. Due to the poor solubility of PSSNH_2_, we could only use dimethyl sulfoxide (DMSO) and dimethylformamide (DMF) which were the only two solvents capable of swelling this parent polymer. Running the reaction in DMF (entry 5), however, led to a significantly lower degree of deamination (47%). We therefore kept DMSO as our solvent for the remainder of the study. Given that deprotonation of the NHC precatalyst is the most important step to initiate the catalytic cycle, we tried three mild bases for this optimization study—K_2_CO_3_ (entry 4), Cs_2_CO_3_ (entry 6), and NEt_3_ (entry 7) affording degrees of deamination of 86%, 30%, and 38%, respectively. The reaction was therefore performed in DMSO with K_2_CO_3_ as the base at 100 °C. At 12 hours (entry 8), the reaction yielded a 55% degree of deamination, which increased to 67% at 18 hours (entry 9), 86% at 24 hours (entry 4), and ultimately 88% at 48 hours (entry 10). Increasing the reaction time from 24 hours to 48 hours only increased the degree of deamination from 86% to 88%. This incremental increase was not deemed sufficient to justify an additional 24 hours of reaction time at the given temperature. We subsequently examined the effects of the temperature of the reaction on the degree of deamination. As indicated in Table 1, the degree of deamination increased proportionally with rising temperature. At 60 °C (entry 11), the degree of deamination was only 33%. However, it increased significantly to 85% at 80 °C (entry 12) and reached 88% at 120 °C (entry 13), slightly surpassing the 86% conversion achieved at 100 °C (entry 4) under the same conditions. This observation prompted us to conduct a second time study at 120 °C. As shown in entries 14 and 15 (Table 1), the degree of deamination was 81% after 12 hours, and reached 86% after 18 hours. Given that the degree of deamination only increased by 2% when the reaction time was extended from 18 hours to 24 hours at 120 °C, we concluded that the optimal reaction conditions were 120 °C for 18 hours with DMSO as the solvent, NHC 4 as the catalyst and K_2_CO_3_ as the base. The sulfinate functionalized PS (86%) was purified using Soxhlet washes in chloroform, methanol, and water.

**Fig. 2:**
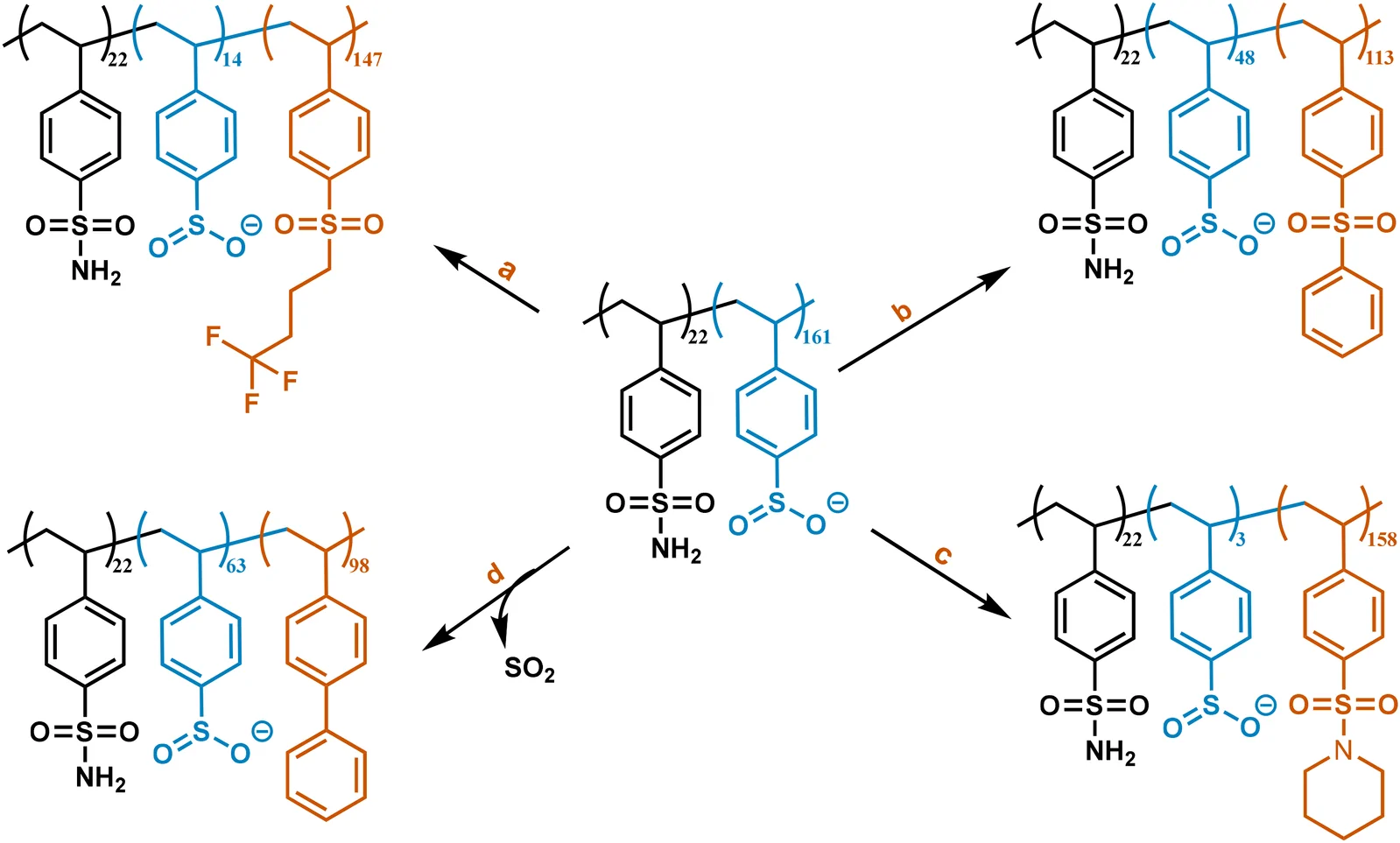
Functionalization of PSSO_2_^−^. Reaction conditions: (a) trifluorobutyl iodide, DMSO, 25 °C, 24 h; (b) tetra-*n*-butylammonium chloride, PhI, Xantphos/Pd, Cs_2_CO_3_, DMSO, 120 °C, 24 h; (c) piperidine, I_2_, H_2_O, 25 °C, 24 h; (d) PhOTf, XPhos/Pd, DMSO, 120 °C, 24 h.

Once the deamination reaction was optimized, we proceeded to investigate the scope of potential functional groups that can be attached onto the phenyl. These functional groups included alkyl sulfones, aryl sulfones, sulfonamides, and aryls (obtained *via* cross-coupling) (Fig. 2). Due to the poor solubility of the resulting polymers, XPS was used throughout to quantify the degree of functionalization (Fig. S5). The conversion calculated using XPS was supplemented with solid-state ^1^H NMR (DF_NMR_) and thermogravimetric analysis (DF_TGA_) (Table 2). The DF_NMR_ was generally slightly lower, but both the DF_NMR_ and DF_TGA_ were within the same range as the ones obtained by XPS. In the paragraphs below, we describe each functional group attachment separately.

**Table 2:** Diversification of sulfinate with various functional group attachments

Entry	Final functional group	Reactants	DF_XPS_ a (%)	DF_NMR_ b (%)	DF_TGA_ c (%)	*T*_d,10%_ d (°C)	Yield (%)
1	Styrene trifluorobutyl sulfone	a: Trifluorobutyl iodide	91	**85**	93	365	85
2	Styrene phenyl sulfone	b: Phenyl iodide	70	**67**	75	328	81
3	Styrene sulfonyl piperidine	c: Piperidine	98	**92**	95	280	82
4	Phenyl styrene	d: Phenyl triflate	74	**70**	N/A	360	75

a Degree of functionalization calculated from XPS.

b Degree of functionalization calculated from ^1^H solid state NMR.

c Degree of functionalization calculated from mass loss *via* TGA.

d Decomposition temperature calculated using TGA.

**Fig. 3:**
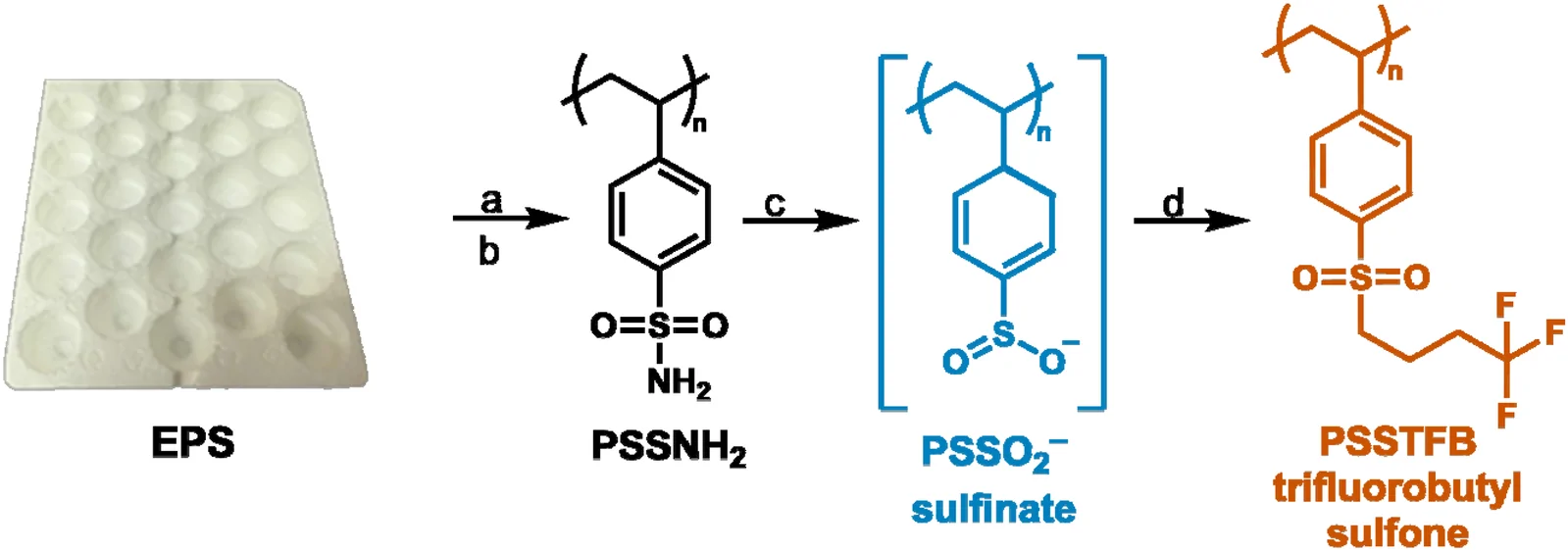
Post-polymerization modification of post-consumer plastic waste, expanded polystyrene (EPS), to polystyrene trifluorobutyl sulfone. Reaction conditions: (a) chlorosulfonic acid, thionyl chloride, CHCl_3_, 25 °C, 8 h; (b) aqueous ammonia, 25 °C, 8 h; (c) benzaldehyde, NHC 4, K_2_CO_3_, DMSO, 120 °C, 24 h; (d) trifluorobutyl iodide, DMSO, 25 °C, 24 h.

To demonstrate reactivity of the sulfinate with electrophilic alkyl iodides, we reacted purified PSSO_2_^−^ with trifluorobutyl iodide to synthesize polystyrene trifluorobutyl sulfone. A solution of trifluorobutyl iodide in DMSO was added to the reaction flask with PSSO_2_^−^ and the reaction was run for 24 hours at 25 °C. The pale-yellow powder thus obtained was purified using Soxhlet wash in chloroform, THF, and water for 24 hours each. The degree of functionalization was calculated to be 91% using the sulfur-to-fluorine ratio (Table 2, entry 1), which was verified by solid state ^1^H NMR where the degree of conversion was calculated to be 85% (Fig. S6). The purity was determined using solid-state ^13^C NMR (Fig. S7) and ^19^F NMR (Fig. S8). The purity of the polymers was verified using solid state ^13^C NMR by overlaying the NMR spectrum of the crude samples and that of the purified functionalized polymers. These experiments showed that sharp signals corresponding to solvent, excess reagents and/or by-products are not present in the polymers purified by Soxhlet washes, and only broad signals characteristic of polymers remain. Given these broad overlapping peaks, however, we cannot confirm the absence of impurities but the ^19^F NMR is pure. We selected the trifluorobutyl group to illustrate that long alkyl chains can be added to the sulfinate and also that it can provide an approach to fluorinate polymers. Additionally, the presence of the fluorine atom facilitated quantification of the degree of conversion by XPS. With efficient functionalization conditions on the model PS, we then moved on to post-consumer expanded polystyrene (EPS) packaging (Fig. 3). The degree of sulfonation and amidation on this plastic waste was calculated to be 91% and 89%, respectively. The polymer was then functionalized with trifluorobutyl iodide under the same reaction conditions as mentioned above to form a fluorinated polymer with the degree of alkylation of 72%.

The sulfinate is also reactive with aryl iodides, therefore PSSO_2_^−^ was reacted with phenyl iodide in the presence of a third generation Buchwald precatalyst, Xantphos/Pd and Cs_2_CO_3_ as the base in DMSO for 24 hours at 120 °C.^45^ The final product was a pale yellow powder which was purified by Soxhlet washes. The degree of functionalization was calculated using the sulfur-to-carbon ratio, where the increase in carbon content compared to the XPS of PSSO_2_^−^ indicated the presence of an additional phenyl group in the polymer. The degree of functionalization was determined to be 70% (Table 2, entry 2). The conversion calculated using XPS was verified using ^1^H NMR to be 67% (Fig. S9). Overall purity of the sample was assessed using solid-state ^13^C NMR (Fig. S10) showing the disappearance of sharp peaks after Soxhlet washing. Sulfinates can also react with amines in the presence of iodine to form sulfonamides by oxidative amination.^46^ Piperidine was chosen as the amine to show that cyclic amines can be used. For the synthesis of polystyrene sulfonyl piperidine (Table 2, entry 3), a solution of piperidine and iodine in water was reacted with PSSO_2_^−^, and the reaction was run for 24 hours at 25 °C. The pale yellow powder was purified by Soxhlet washes. The degree of functionalization, calculated using the sulfur-to-nitrogen ratio, was found to be 98%. This was cross-verified using ^1^H NMR to be 92% (Fig. S11), demonstrating that high degrees of amination are achievable. As before, we confirmed that small molecule impurities were washed off by solid-state ^13^C NMR (Fig. S12). Lastly, we demonstrated a C–C cross-coupling reaction between the sulfinate and an aryl triflate. We used phenyl triflate in the presence of a second-generation Buchwald precatalyst, XPhos-Pd-G2, in DMSO for 24 hours at 120 °C to form polyphenyl styrene, yielding a styrene polymer functionalized with phenyl group at the *para* position and sulfur dioxide as a byproduct.^47^ The decrease in the sulfur-to-carbon ratio in the XPS of the final polymer compared to that of PSSO_2_^−^ suggested partial desulfonation and cross-coupling, with the degree of functionalization calculated to be 74%, as verified by ^1^H NMR giving a conversion of 70% (Fig. S13). Solid state ^13^C NMR (Fig. S14) showed that no sharp peaks were left in the sample post washing. The examples above highlight the potential of using sulfinates as lynchpins for the functionalization of PS. We expect that other alkyl and aryl iodides, amines, and aryl triflates could be used to access a variety of functional groups.

**Fig. 4:**
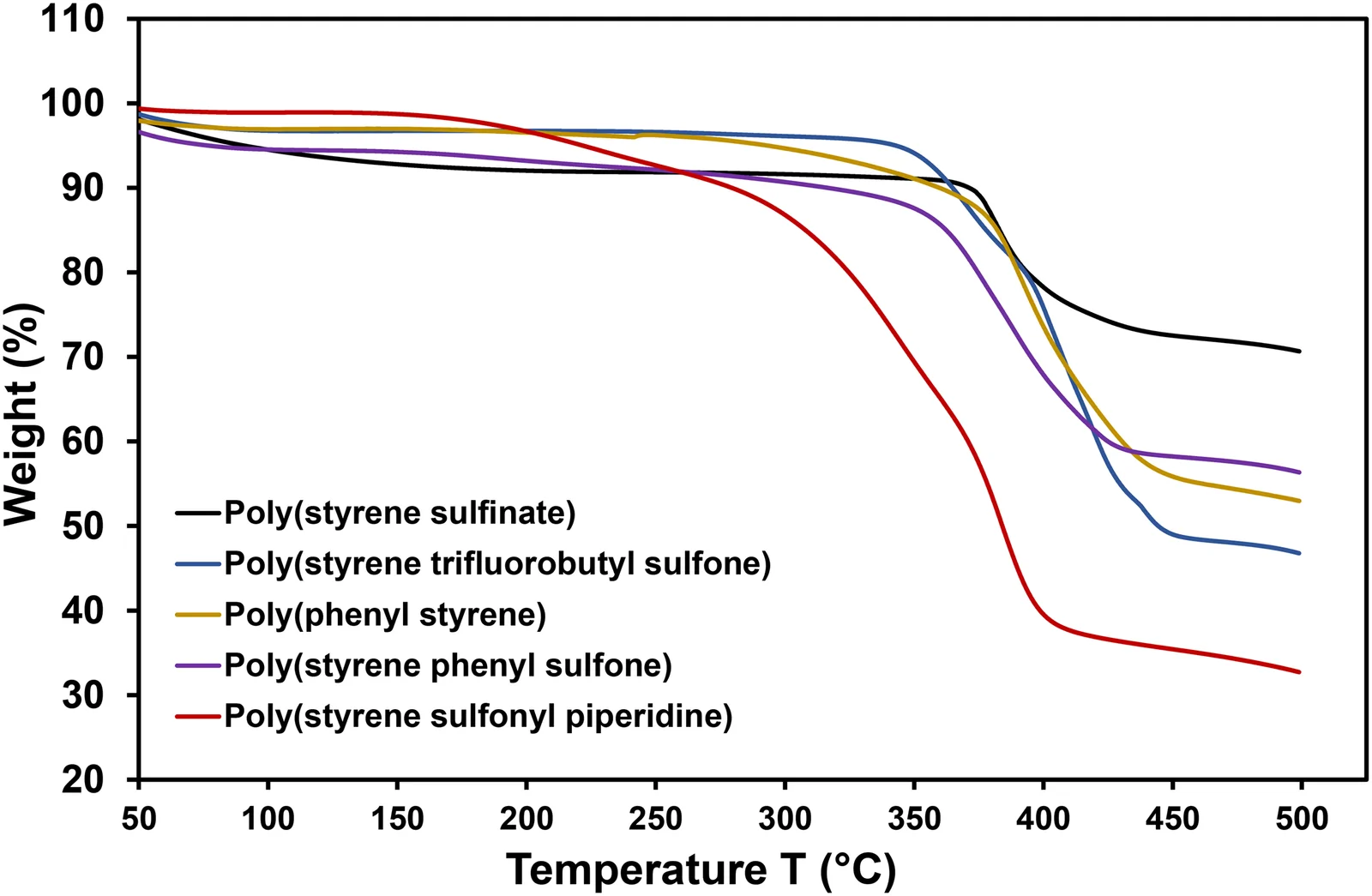
TGA traces of the polymers functionalized from poly(styrene sulfinate).

While the functionalized polymers were not soluble in any of the common and uncommon solvents, the polymers did swell up in DMSO, and hence we could hot-press the polymers into film-like structures, as shown in Fig. S15. For some materials, such as polystyrene sulfonamide, polystyrene phenyl sulfone, and polystyrene sulfonyl piperidine, it worked well, and we obtained free-standing films. In contrast, others, such as polystyrene trifluorobutyl sulfone and polyphenyl styrene, were too brittle even though they seemed moldable. We then assessed the thermal stability of the modified polymers using thermogravimetric analysis (TGA), as shown in Fig. 4. Poly(styrene sulfinate) (PSSO_2_^−^) exhibited a decomposition temperature of 372 °C. In comparison, the functionalized polymers showed lower degradation temperatures (Table 2). Poly(styrene trifluorobutyl sulfone) degraded at 365 °C (entry 1), poly(styrene phenyl sulfone) at 328 °C (entry 2), polystyrene sulfonyl piperidine at 280 °C (entry 3), and poly(phenyl styrene) at 360 °C (entry 4). The mass loss for entries 1–3 approximately corresponded to the mass attributed to the functionalized sulfone moieties, supplementing the degree of functionalization calculated using XPS. However, for poly(phenyl styrene) (entry 4), the degradation likely followed a different pathway, preventing direct verification of the degree of cross-coupling. These results demonstrate that the sulfinate functionality can serve as a versatile lynchpin for post-polymerization diversification. The distinct thermal stabilities of the resulting polymers highlight the ability to tune material properties through PPM.

## Conclusions

We successfully developed a synthetic approach to modify polystyrene to a reactive polystyrene sulfinate by organocatalyzed deamination, using an N-heterocyclic carbene. This transformation efficiently converted polystyrene sulfonamide into a sulfinate intermediate with degrees of deamination over 85%. This reactive intermediate was then treated with a broad range of functional groups, including alkyl iodides, aryl iodides, amines, and aryl triflates, leading to degrees of functionalization between 74% and 98%. These polymers exhibited distinct chemical and thermal properties from the parent polystyrene, demonstrating the potential of the transformation in post-polymerization modification. This study highlights sulfinate intermediates as a versatile lynchpin to create a diverse library of functionalized styrenic polymers. The transformation was also successfully applied to post-consumer plastic waste, achieving a 72% alkylation with a trifluorobutyl group. Overall, this method proves to be a robust tool for post-polymerization modification, efficiently attaching new functional groups and yielding a range of functionalized polymers with potential applications in hydrophobic, antibacterial, or flame-retardant materials.

## Author contributions

T. S. and L. V. K. conceived the project idea and designed the experiments. T. S. and D. M. N synthesized the polystyrene and functionalized the polystyrene to poly(styrene sulfonamide) analyzed them. V. S. D. performed the GPC experiments and helped T. S. analyze the data. T. S. ran optimization studies for synthesis of poly(styrene sulfinate) with the help of H. G. T. S. conceived and designed the functionalization of poly(styrene sulfinate) and ran the XPS and analyzed the data. C. M. Q. ran the solid-state NMR and helped T. S. analyze the data. L. V. K. provided guidance and funding. T. S. and L. V. K. wrote the manuscript.

## Conflicts of interest

The authors declare no competing financial interest.

## Supplementary Material

PY-017-D5PY01024A-s001

## Data Availability

The data for this article is available freely in the Harvard Dataverse public repository at: https://doi.org/10.7910/DVN/OT2MAH. Data available: raw GPC of the model polystyrene, IR spectra, thermogravimetric analysis (TGA), NMR spectra files, and X-ray photoelectron spectroscopy of survey spectra and relevant individual elements. Processed data is available in the supplementary information (SI). Supplementary information: GPC trace of the model polystyrene, IR spectra, thermogravimetric analysis (TGA) traces, NMR spectra, and X-ray photoelectron spectroscopy of survey spectra and relevant individual elements. See DOI: https://doi.org/10.1039/d5py01024a.
