# Supplementary material for: Organo-catalyzed deamination of polystyrene sulfonamide for diverse post-polymerization modification of styrenic polymers
Source: Polym Chem. 2026 Apr 1;17(17):1721–9. doi: 10.1039/d5py01024a (PMC13058796; doi:10.1039/d5py01024a)
Supplement: PY-017-D5PY01024A-s001 [file PY-017-D5PY01024A-s001.pdf]

Supplementary Information (SI) for Polymer Chemistry.
This journal is © The Royal Society of Chemistry 2026

Supplementary Information for

Organo-Catalyzed Deamination of Polystyrene Sulfonamide for Diverse Post-Polymerization Modification of Styrenic Polymers

Tulaja Shrestha,[1] Dan My Nguyen,[1] Harrison Goehrig,[1] Vidhika S. Damani,[2] Caitlin Quinn,[1] Laure V. Kayser[1,2*]

[1]Department of Chemistry and Biochemistry, University of Delaware, Newark, DE 19711, United States

[2]Department of Materials Science and Engineering, University of Delaware, Newark, DE 19711, United States

*Corresponding author: lkayser@udel.edu

General

I. Materials

Calcium hydride (CaH_2), *n*BuLi, chlorosulfonic acid, thionyl chloride, aqueous NH_3, 3-ethyl-5-(2-hydroxyethyl)-4-methyl-1,3-thiazolium bromide (NHC 1), 1,4-N,N-diphenyl-3-phenyl triazol-1-ium tetrafluoroborate (NHC 2), 2-pentafluorophenyl-6,7-dihydro-5H-pyrrolo[2,1-c][1,2,4]triazol-2-ium tetrafluoroborate (NHC 3), 2-Mesityl-2,5,6,7-tetrahydropyrrolo [2,1-c] [1,2,4]triazol-4-ium chloride (NHC 4), benzaldehyde, potassium carbonate (K_2CO_3), cesium carbonate (Cs_2CO_3), triethyl amine (NEt_3), trifluorobutyl iodide, imidazole, iodine, phenyl iodide, tetra-n-butylammonium chloride), Xantphos-Pd-G3 precatalyst, phenyl triflate, XPhos-Pd-G2 precatalyst

were purchased from Sigma Aldrich and used as received. Methanol (MeOH), chloroform ($CHCl_3$), anhydrous dimethyl sulfoxide (DMSO), and anhydrous dimethyl formamide (DMF) were bought from Fisher Scientific and used as received. Cyclohexane was purchased from Fisher Scientific, dried in CaH_2, and distilled into a two-necked round bottom flask (RBF) under vacuum for use. Styrene was purchased from Sigma Aldrich and was purified by passing it through a silica column and used immediately.

II. Characterization Methods

X-ray photoelectronic spectroscopy (XPS): The data for XPS were acquired using a K-Alpha XPS from Thermo Fisher Scientific.

Nuclear magnetic resonance (NMR): ^1H NMR spectra were obtained on a Bruker 600 MHz spectrometer. All the crude reaction mixtures and polymers were dissolved in $CDCl_3$ at a concentration of ~10 mg/mL. For solid-state NMR, the spectra were taken on a AVIIIS 600 MHz spectrometer. All measurements were taken at room temperature.

Solid state NMR spectra were acquired on a Bruker Avance III 600 MHz NMR spectrometer with a 3.2mm HX or HCN probe at spinning speeds of 20 or 22 kHz and frequencies of 600.393 MHz, 564.894 MHz, 150.983 MHz, and 60.841 MHz for ^1H, ^{19}F, ^{13}C, and ^{15}N respectively. ^{13}C direct polarization spectra utilized a 3.3 μs 90° pulse, 125-250s recycle delay, and 416-1024 scans were averaged. Recycle delays for ^{13}C spectra were set to $5T_1$ as determined for each sample using the method of Torchia.[1] ^{15}N spectra utilized ^1H-^{15}N cross polarization (CP), a 3s recycle delay, and 32,768 scans were averaged. 75 kHz ^1H SPINAL-64 decoupling was applied during acquisition of ^{13}C and ^{15}N spectra.[2] ^{19}F spectra used a 2.96 μs 90° pulse, 256 scans per spectrum, and 2-3s recycle

delay, which was confirmed to be >5T_1 with an inversion recovery T_1 relaxation measurement for each sample. ^{1}H spectra used a 2.45 μs 90° pulse, 32 scans per spectrum, and 10s recycle delay, which was confirmed to be >5T_1 with an inversion recovery T_1 relaxation measurement for each sample. Some ^{1}H and ^{1}H-^{15}N CP spectra for PSSTFB were acquired on a Bruker Avance III 850 MHz NMR spectrometer with a 3.2mm HCN probe at a spinning speed of 22 kHz, 10s recycle delay, and 32 scans (^{1}H) or 14 kHz spinning, 2s recycle delay, and 81,920 scans (15N). **Gel permeation chromatography (GPC):** GPC was performed using a Tosoh EcoSEC 8320 (Tosoh Bioscience) system running in OPTIMA™ THF, using a TSKgel HHR-H guard column and two TSKgel GMHHR-N columns in series, and a refractive index detector. Number-average (M_n) and weight-average (M_w) molecular weights and dispersity (*Đ*) were determined by calibration against narrow dispersity polystyrene standards (Tosoh Bioscience, PStQuick C and PstQuick D).

Fourier transform infrared spectroscopy (FTIR): All measurements were conducted on a PerkinElmer spectrum 100 FTIR spectrometer. FTIR spectra were recorded from 4000 cm^{-1} to 600 cm^{-1}.

Thermogravimetric Analysis (TGA): All measurements were conducted on a TA Instruments Discovery Series Thermogravimetric Analyzer. The temperatures were recorded from 20 °C to 500 °C with with a heating rate of 10 °C min^{-1} under nitrogen.

Procedure for the anionic polymerization of polystyrene (PS)

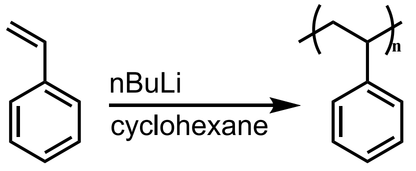

Glassware was dried in a vacuum oven overnight. About 300 mL of cyclohexane (0.5 M) was then cannula transferred to a 500 mL three-necked RBF. 50 mL of styrene (0.44 mol) was injected into the reaction vessel, followed by 3.5 mL of 1.6 M *n*BuLi (4.5 mmol, 0.01 equiv.). The reaction gradually turned deep orange and was run for 1 h. After the reaction was completed, the reaction vessel was exposed to air, and 50 mL MeOH was added while stirring the reaction to ensure the termination of the living polymerization. This solution was then concentrated using a rotatory evaporator, and the final solution was precipitated in MeOH to obtain white powder. The powder was dissolved in acetone and precipitated in MeOH twice, which resulted in a fine white powder of polystyrene (39 g, 87% yield). ^{1}H NMR (600 MHz, $CDCl_3$) δ 7.1 (s, 2H), 6.8 (s, 3H), 1.7 (s, 3H). GPC: M_n = 19 kDa, $Đ$ = 1.2.

III. Procedure for synthesis of polystyrene sulfonamide ($PSSNH_2$)

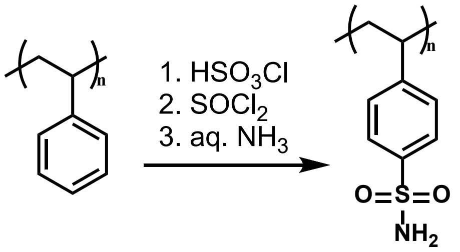

In ambient conditions, previously synthesized polystyrene (10 g, 96 mmol) was added to a two-necked RBF. 250 mL of chloroform was added to dissolve the powder. The reaction vessel was sealed, and chlorosulfonic acid (HSO_3Cl) (8 mL, 120 mmol) was added dropwise over the course of 10 minutes. Yellow globs precipitated from the reaction solution. The reaction was run for 4 hours, followed by adding thionyl chloride ($SOCl_2$) (8.2 mL, 120 mmol). This process was run for 6 hours. After the completion of the reaction, the system was purged with N_2 for 30 minutes to capture any HCl produced during the chlorosulfonation process. The solid was filtered and purified through Soxhlet washes in $CHCl_3$, MeOH, and H_2O for 12 hours each. The brown solid was dried and characterized to ensure the presence of chloride. The conversion to polystyrene sulfonyl chloride was verified using FTIR where the presence of the S-Cl peak at 1360 cm^{-1} confirms the formation of the bond (**Figure S1**). The resulting solid was added to an RBF, and 75 mL of 30% aqueous NH_3 was added to the reaction flask. This reaction was run for 6 hours in a heterogenous mixture, and the solid was filtered and purified through Soxhlet wash in $CHCl_3$, MeOH, and H_2O for 12 hours each (procedure adapted from *European Polymer Journal* **1991**, *27* (6), 461–463). This afforded a pale yellow solid (15 g, 79% yield). Solid state ^{13}C NMR (800 MHz) δ 149.67 (s, 1C), 139.58 (s, 1C), 126.22 (s, 4C), 40.85 (s, 2C); ^{15}N NMR (800 MHz) δ 94.18 (s, 2N). XPS: degree of sulfonation: 95%, degree of amidation: 88%.

IV. General procedure for the optimization of the deamination reaction

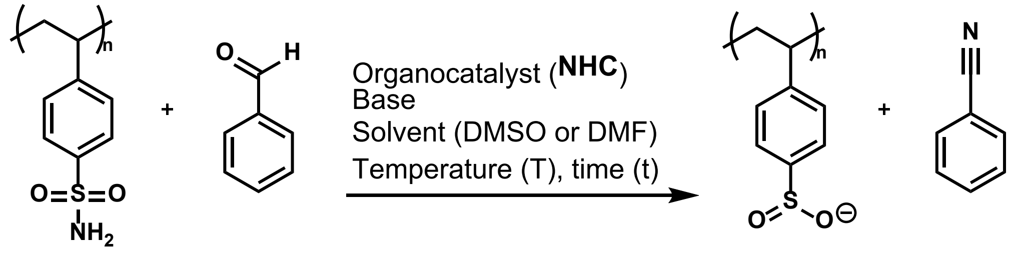

In a 20 mL scintillation vial with a septum cap, NHC (10 mol%) was added, followed by $PSSNH_2$ (1.0 equiv) and the selected base (3 equiv). The vial was sealed and degassed for 20 minutes under nitrogen, then benzaldehyde (2 equiv) was added to the reaction vials, followed by 9 mL (0.1 M) of anhydrous solvent. The reaction was run at various temperatures and times, as stated in **Table 1**. Once the process was completed, the solid was separated from the mixture through filtration. Soxhlet washes were performed in $CHCl_3$, MeOH, and water for 12 hours each for purification (procedure adapted from *Journal of the American Chemical Society* **2019**, *141* (4), 1441–1445). The powders were dried off in a vacuum oven for 24 hours, and the degree of deamination was determined using XPS.

V. Scale-up synthesis of polystyrene sulfinate ($PSSO_2^-$) from $PSSNH_2$ with optimized reaction conditions

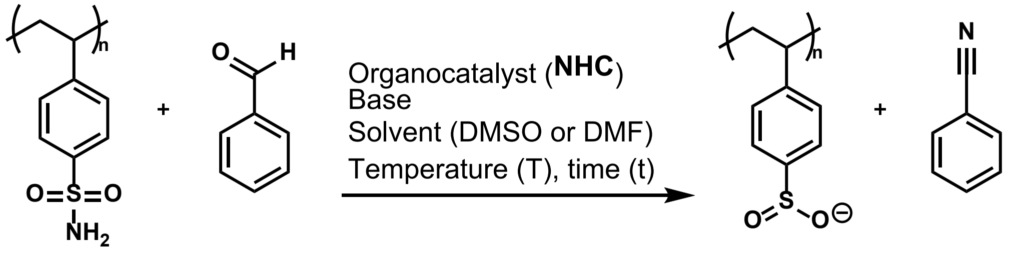

In a 250 mL round bottom flask, NHC 4 (482.7 mg, 10 mol%) was added, followed by $PSSNH_2$ (3.2 g, 18.3 mmol) that was previously synthesized, and K_2CO_3 (7.6 g, 54.9 mmol). Benzaldehyde (3.7 mL, 36.6 mmol) was added to the flask, followed by anhydrous DMSO (183 mL, 0.1 M). The reaction was run for 18 hours at 120 °C. The reaction was allowed to cool, and the solid was

separated from the mixture through filtration, and the bases were washed off with water for 3 hours. Soxhlet washes were performed in $CHCl_3$, MeOH, and water for 12 hours each for purification (procedure adapted from *Journal of the American Chemical Society* **2019**, *141* (4), 1441–1445). The powder was dried off in a vacuum oven for 24 hours. (2.49 g, 82% yield). The degree of deamination of the dried powder (2.49 g, 82% yield) was quantified to be 86% using XPS using the atomic ratio of sulfur: nitrogen in the sample.

VI. Functionalization of $PSSO_2^-$ with trifluorobutyl iodide

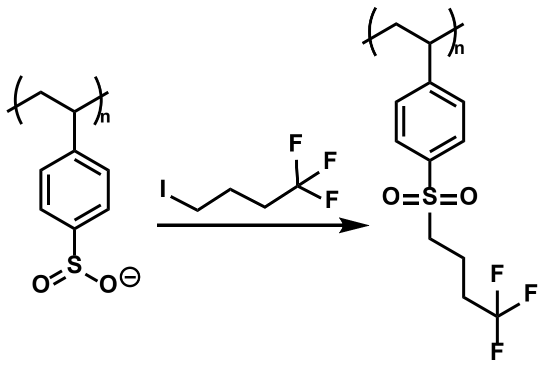

In a 20 mL scintillation vial, $PSSO_2^-$ (149.7 mg, 0.9 mmol) was added followed by anhydrous DMSO (4.4 mL, 0.05 M). After allowing this reaction mixture to chill to 0 °C for 30 minutes, a freshly prepared solution of trifluorobutyl iodide (170 µL, 1.3 mmol) in DMSO (4.4 mL, 0.75 M) was added dropwise and the ice bath was removed to allow the reaction to equilibrate at room temperature at 25 °C. The mixture was stirred for 24 hours and the resulting solid was separated from the mixture through filtration. Soxhlet washes with $CHCl_3$ and MeOH were performed for 12 hours each to remove excess iodide and remaining DMSO. The powder was dried off in vacuum oven for 24 hours. The degree of alkylation of the dried powder (201.1 mg, 85% yield) was quantified to be 91% using XPS by using the atomic ratio of sulfur: fluorine in the sample. Solid

state ^{13}C NMR (800 MHz) δ 142.39 (s, 2C), 126.42 (s, 4C), 41.06 (s, 6C); ^{19}F NMR (800 MHz) δ −65.48 (s, 3F). XPS: degree of functionalization: 91%.

VII. Functionalization of $PSSO_2^-$ with phenyl iodide

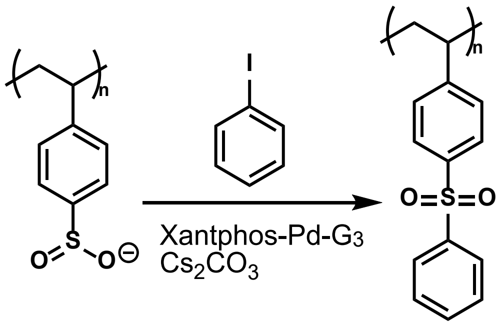

This reaction was performed in a glovebox environment under nitrogen. In a 20 mL scintillation vial, PSS (149.7 mg, 0.9 mmol), tetra-N-butylammonium chloride (293.5 mg, 1.1 mmol), iodobenzene (215.4 mg, 1.1 mmol), Xantphos-Pd-G3 precatalyst (83.5 mg, 0.09 mmol), and Cs_2CO_3 (430.1 mg, 1.3 mmol) were added followed by anhydrous DMSO (8.8 mL, 0.1 M). The mixture was heated at 120 °C for 24 hours. The product was separated from the mixture through filtration after completion of the reaction. Soxhlet washes with ethyl acetate, chloroform, and water were performed for 24 hours to remove the Pd catalyst and excess reagents (procedure adapted from *The Journal of Organic Chemistry* **2004**, *69* (17), 5608–5614). The powder was dried off in a vacuum oven for 24 hours. The degree of functionalization of the dried powder (168.2 mg, 81% yield) was quantified to be 70% using XPS by using the atomic ratio of sulfur: carbon in the sample. Solid state ^{1}H NMR (800 MHz) δ 6.39 (s, 9H), 3.00 (s, 1H), 0.8(s, 1H); ^{13}C NMR (800 MHz) δ 145.83 (s, 9C), 125.70 (s, 3C), 41.04 (s, 2C); XPS: degree of functionalization: 70%.

VIII. Functionalization of $PSSO_2^-$ with piperidine

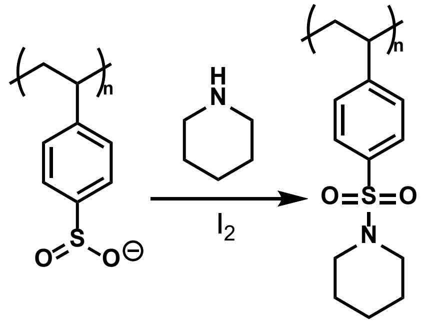

In a 20 mL scintillation vial, PSS (149.7 mg, 0.9 mmol) was added followed by water (8.8 mL, 0.1 M). Piperidine (130 µL, 1.3 mmol) was added, followed by iodine (223.4 mg, 0.9 mmol). The mixture was stirred vigorously at room temperature for 24 hours. The product was separated from the mixture through filtration after completion of the reaction. Soxhlet wash with water was performed for 18 hours to remove excess iodide and piperidine (procedure adapted from *Green Chemistry* **2015**, *17* (3), 1400–1403). The powder was dried off in a vacuum oven for 24 hours. The degree of alkylation of the dried powder (176.1 mg, 82% yield) was quantified to be 98% using XPS by using the atomic ratio of sulfur: nitrogen in the sample. Solid state ^{1}H NMR (800 MHz) δ 7.96 (s, 4H), 4.33 (s, 4H), 3.39 (s, 1H), 1.59 (s, 9H); ^{13}C NMR (800 MHz) δ 146.45 (s, 2C), 127.69 (s, 4C), 45.71 (s, 2C), 40.56 (s, 1C), 24.13 (s, 4C); XPS: degree of functionalization: 98%.

IX. Functionalization of $PSSO_2^-$ with phenyl triflate

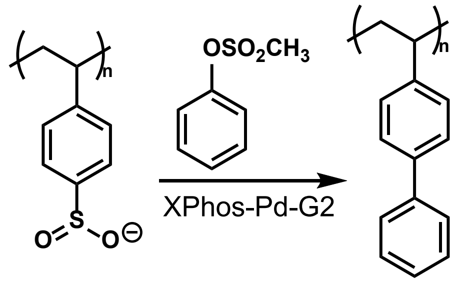

This reaction was performed in a glovebox environment under nitrogen. In a 20 mL scintillation vial, PSS (149.7 mg, 0.9 mmol) was added followed by anhydrous DMSO (8.8 mL, 0.1 M). Phenyl triflate (171 µL, 1.1 mmol) and XPhos-Pd-G2 precatalyst (69 mg, 10 mol%) were added and the mixture was heated at 120 °C for 24 hours. The product was separated from the mixture through filtration after completion of the reaction. Soxhlet washes with ethyl acetate and water were performed for 24 hours to remove the Pd catalyst and excess triflate (procedure adapted from *The Journal of Organic Chemistry* **2012**, *77* (22), 10468–10472). The powder was dried off in a vacuum oven for 24 hours. The degree of functionalization of the dried powder (120.4 mg, 75% yield) was quantified to be 74% using XPS by using the atomic ratio of sulfur: carbon in the sample. Solid state ^{1}H NMR (800 MHz) δ 8.74 (s, 5H), 5.26 (s, 4H), 2.25 (s, 3H); ^{13}C NMR (800 MHz) δ 146.81 (s, 3C), 127.32 (s, 9C), 42.29 (s, 1C), 32.87 (s, 1C); XPS: degree of functionalization: 61%.

X. Hot-pressing the functionalized polymers

15 mg of each polymer sample (polystyrene sulfonamide, polystyrene trifluorobutyl sulfone, polystyrene phenyl sulfone, polystyrene sulfonyl piperidine, and polyphenyl styrene were added to 1 dram glass vials, followed by the addition of 3 mL anhydrous DMSO**.** The vials were sealed and heated at 150 °C for 48 h to allow the polymers to swell. The resulting swollen materials were transferred between two pre-cleaned glass microscope slides and compressed using calipers to

maintain uniform pressure across the sample. The glass-slide assemblies were placed in an oven and heated at 120 °C for 12 h to remove residual solvent and promote film formation. After cooling to room temperature, the slides were separated to release the polymer films. Polystyrene sulfonamide, polystyrene phenyl sulfone, and polystyrene sulfonyl piperidine formed continuous, cohesive films, whereas polystyrene trifluorobutyl sulfone and polyphenyl styrene produced fibrous films under identical conditions.

XI. Supplementary figures

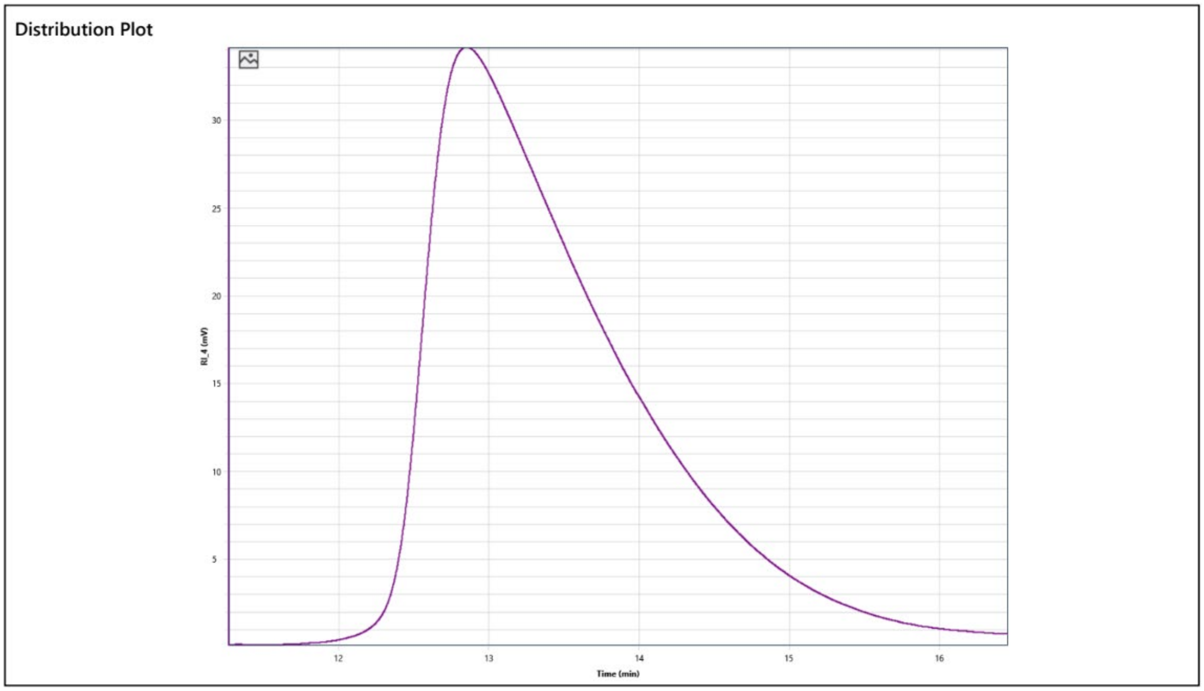

Figure S1: GPC chromatogram of model polystyrene (PS).

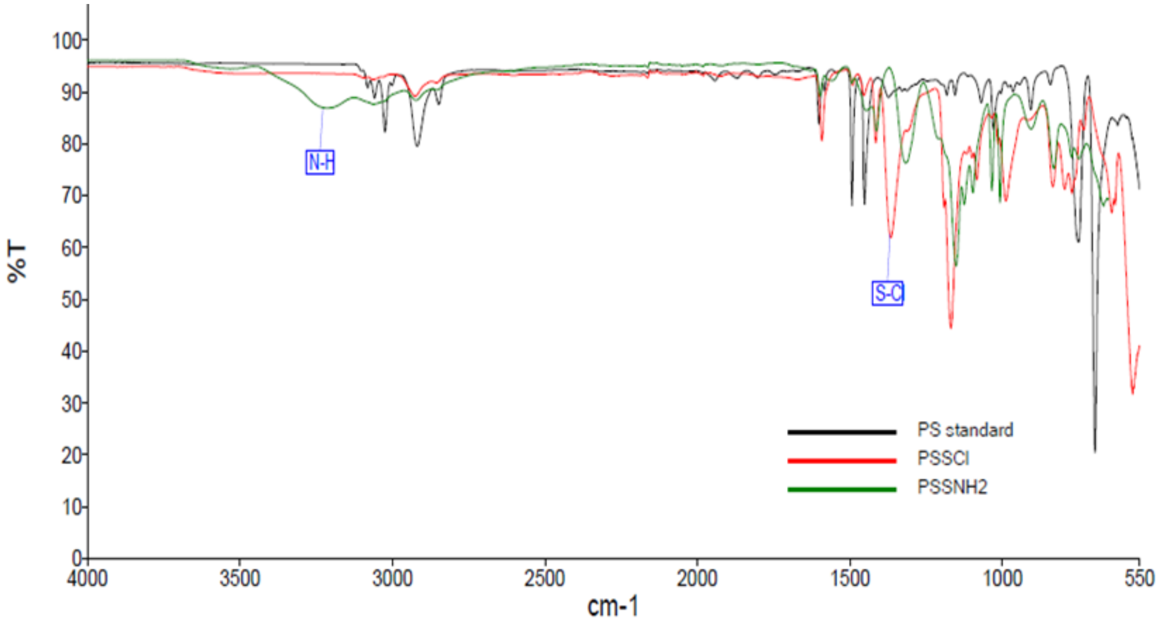

Figure S2: FTIR of PS (black spectrum), PS-sulfonyl chloride (red spectrum), and $PSSNH_2$ (green spectrum) showing the presence of S-Cl group in 1360 cm^{-1} in the red spectrum followed by its absence in the green spectrum and emergence of the N-H peak at 3200 cm^{-1}

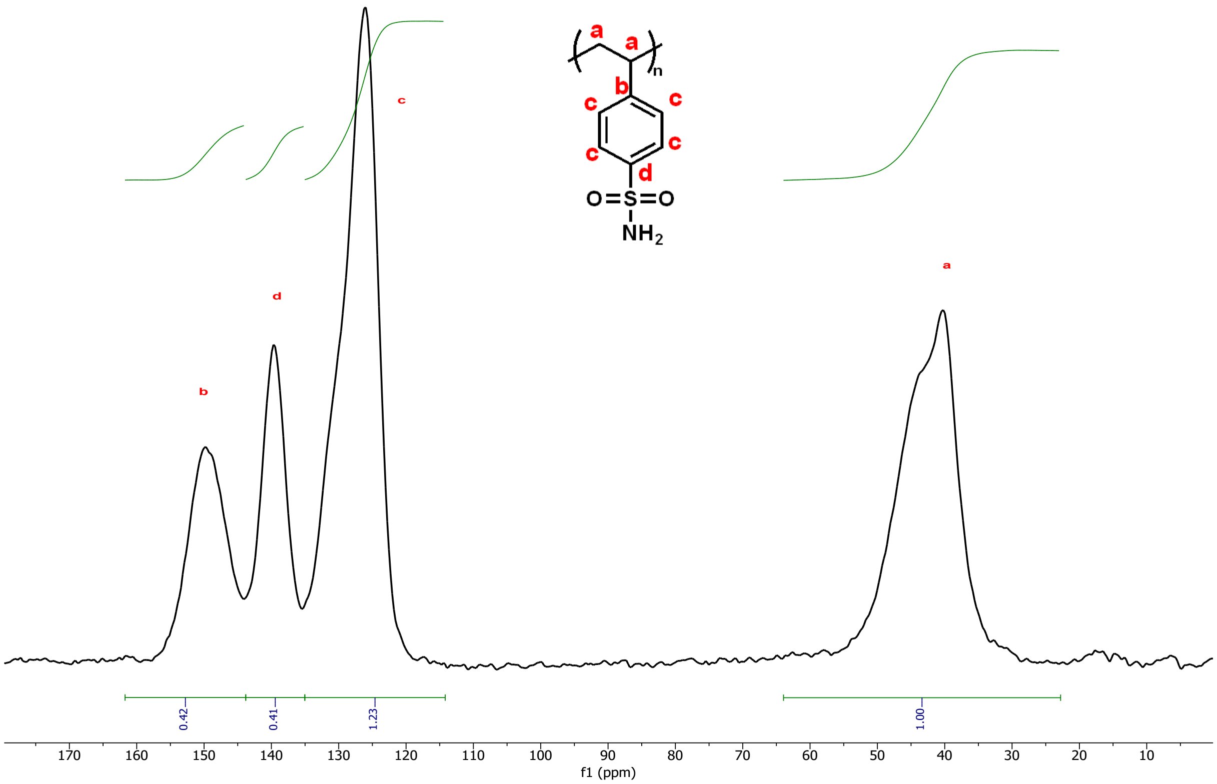

Figure S3. Cross-polarized solid-state ^{13}C NMR spectrum of polystyrene sulfonamide.

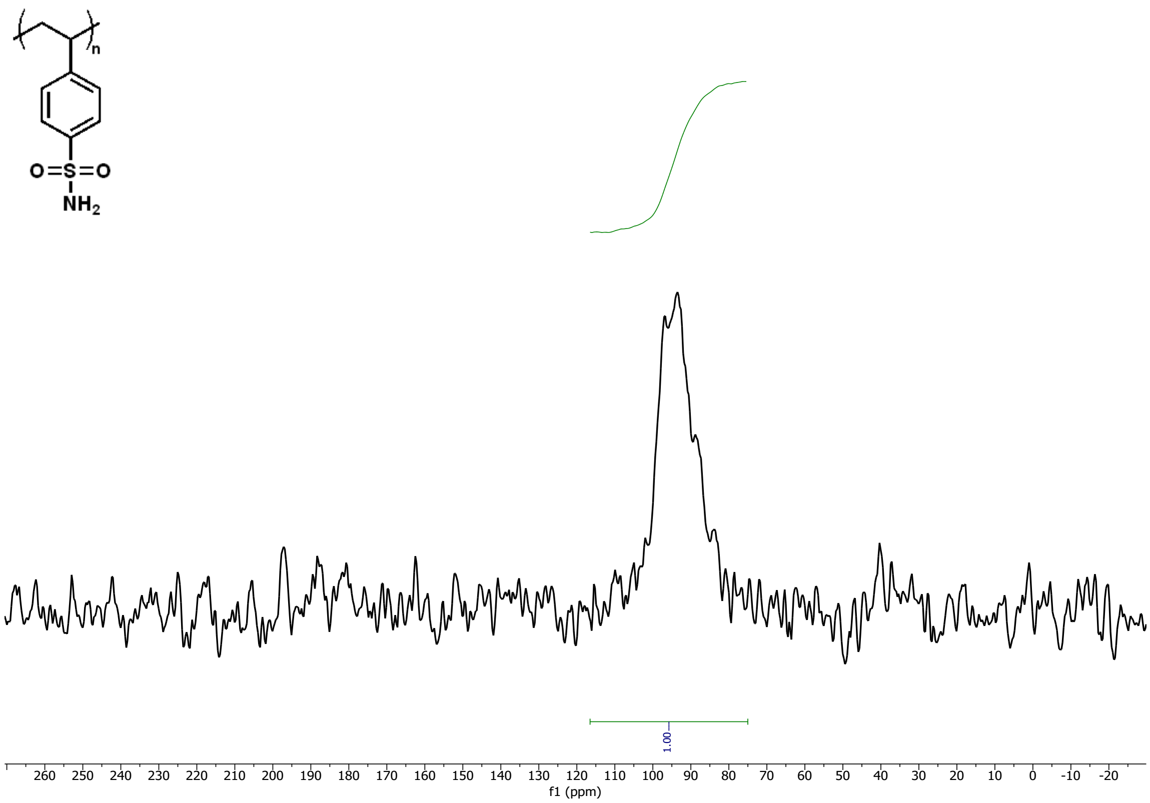

Figure S4. Cross-polarized solid-state ^{15}N NMR spectrum of polystyrene sulfonamide.

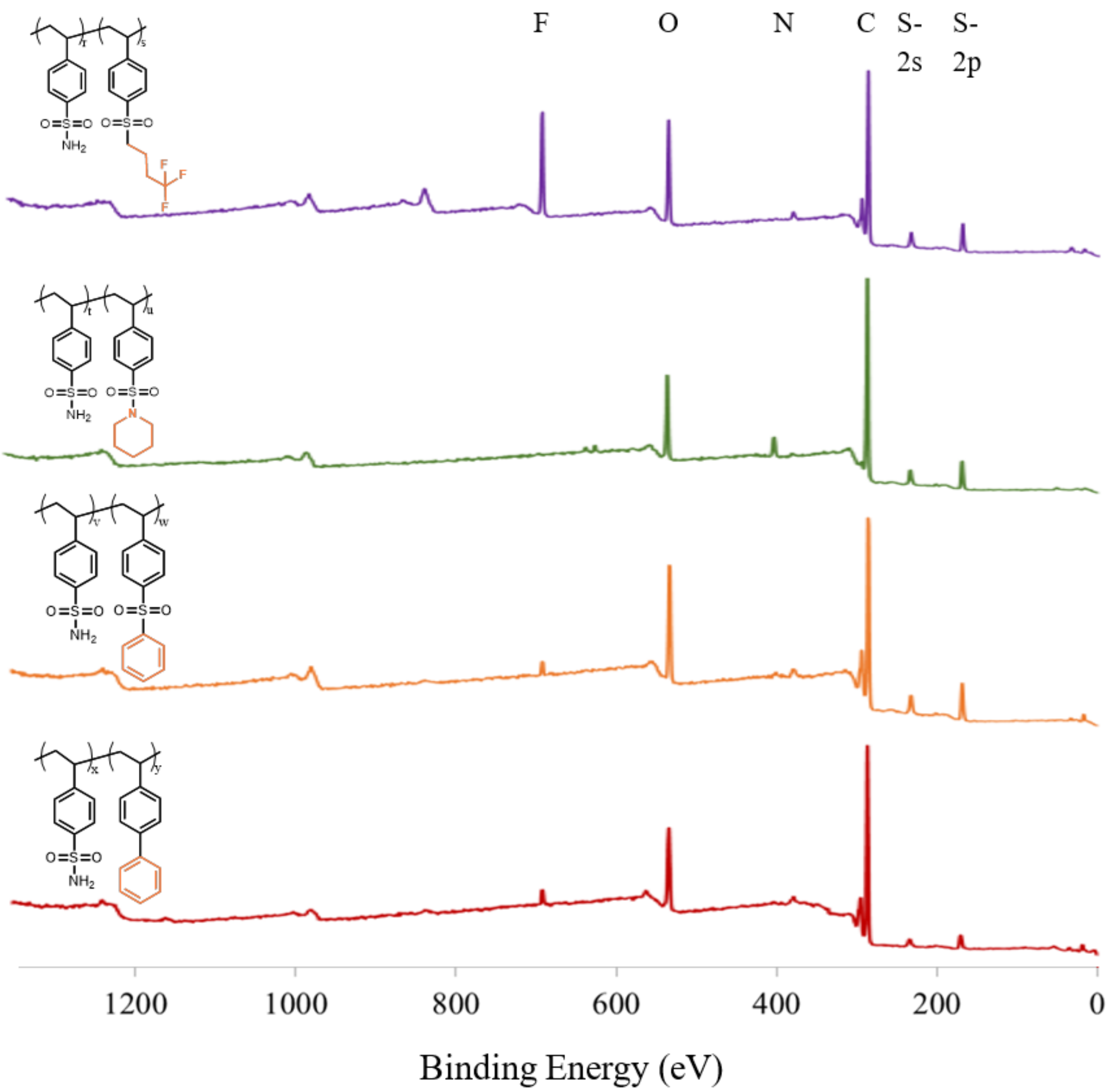

Figure S5. XPS spectra of the functionalized polymers with the elements listed above their respective peak ranges

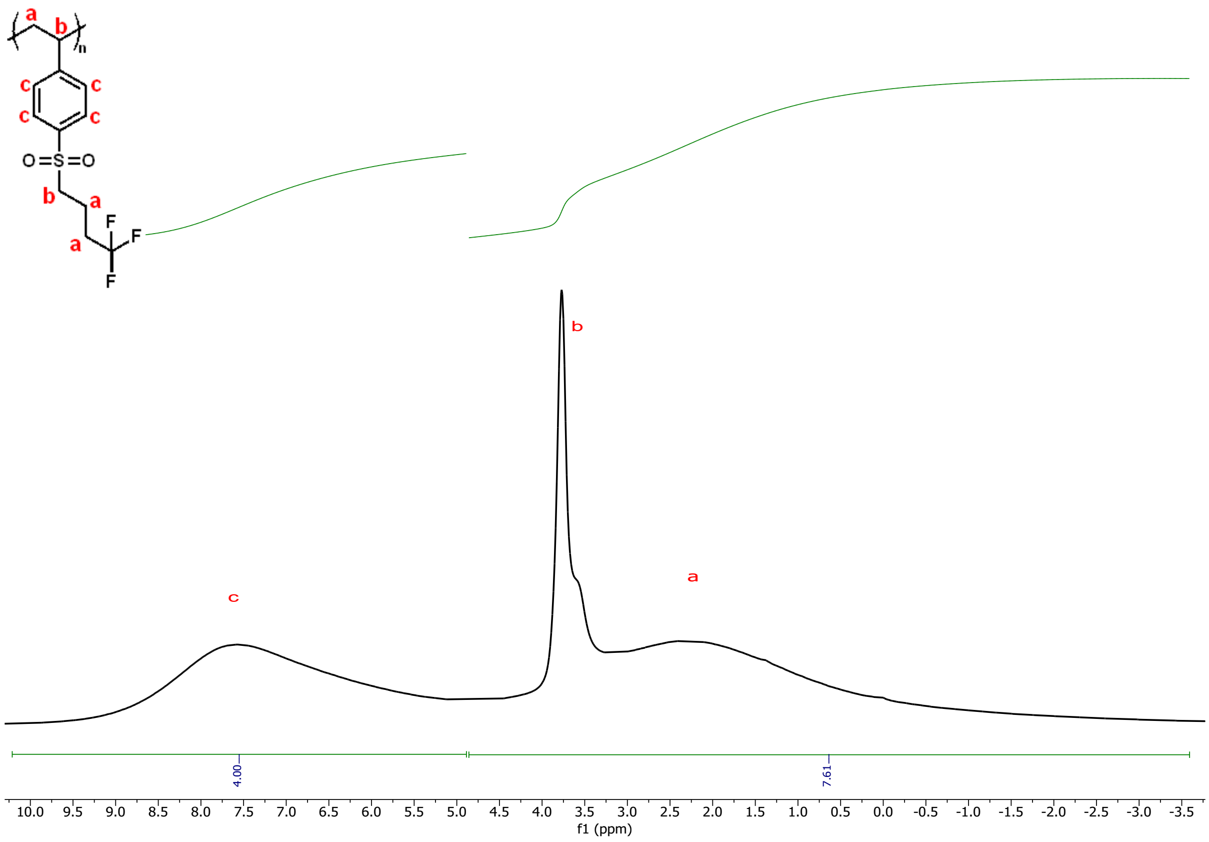

Figure S6. Solid-state ^{1}H NMR spectrum of the polystyrene trifluorobutyl sulfone

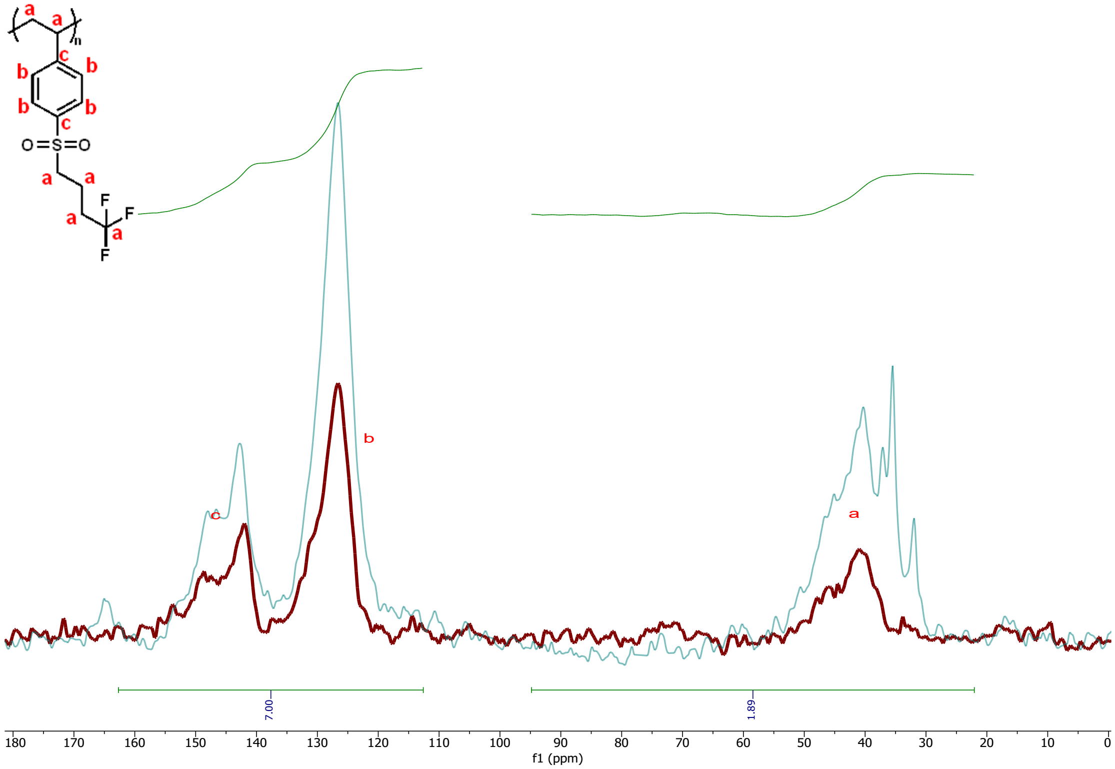

Figure S7. Direct-polarization solid-state ^{13}C NMR spectrum of the polystyrene trifluorobutyl sulfone. The red and green spectra represent purified and crude samples, respectively.

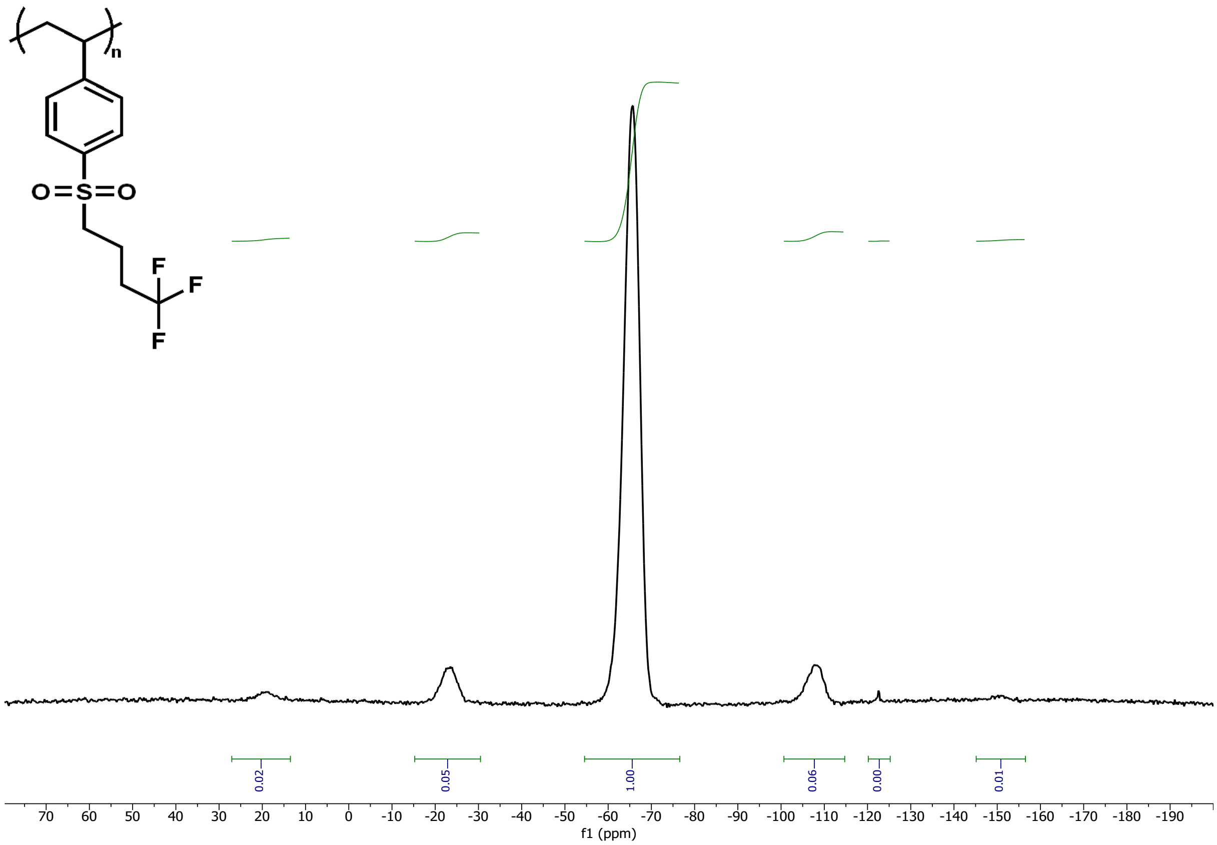

Figure S8. Solid-state ^{19}F NMR spectrum of the polystyrene trifluorobutyl sulfone

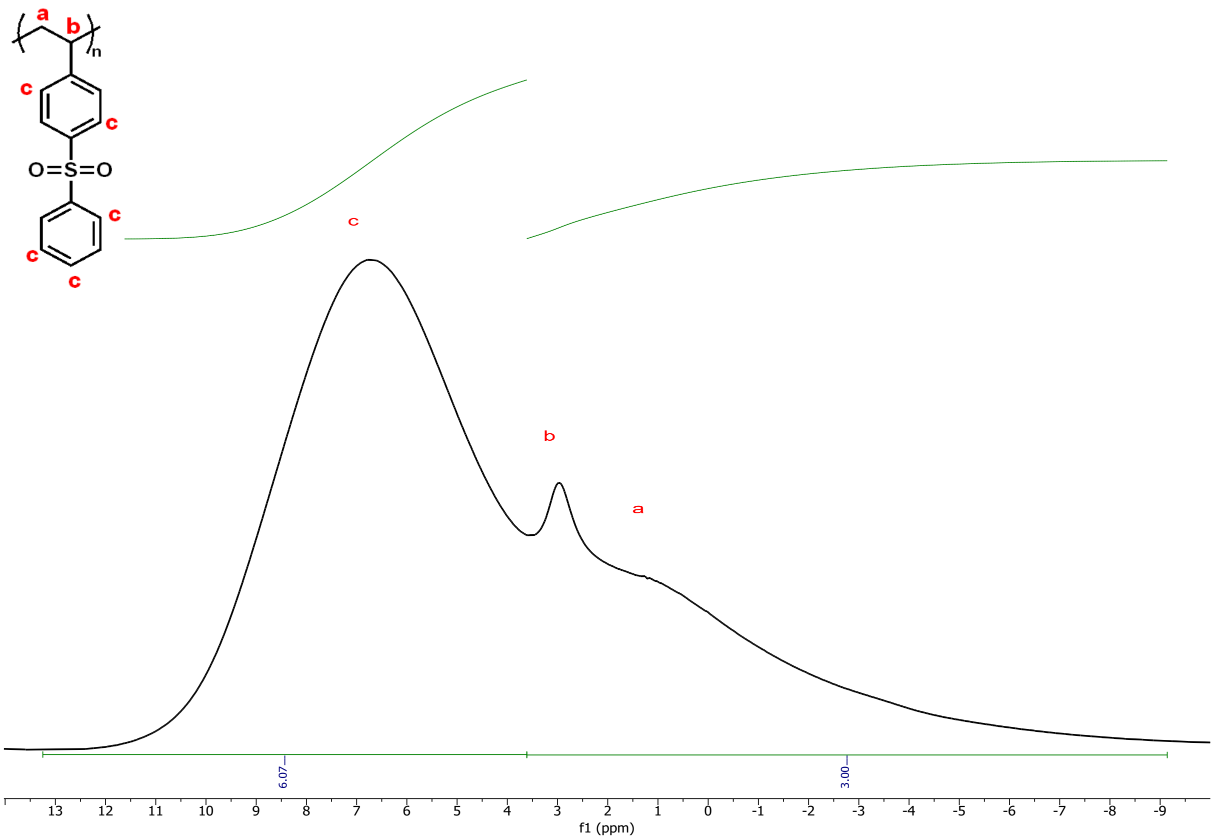

Figure S9. Solid-state ^{1}H NMR spectrum of the polystyrene phenyl sulfone

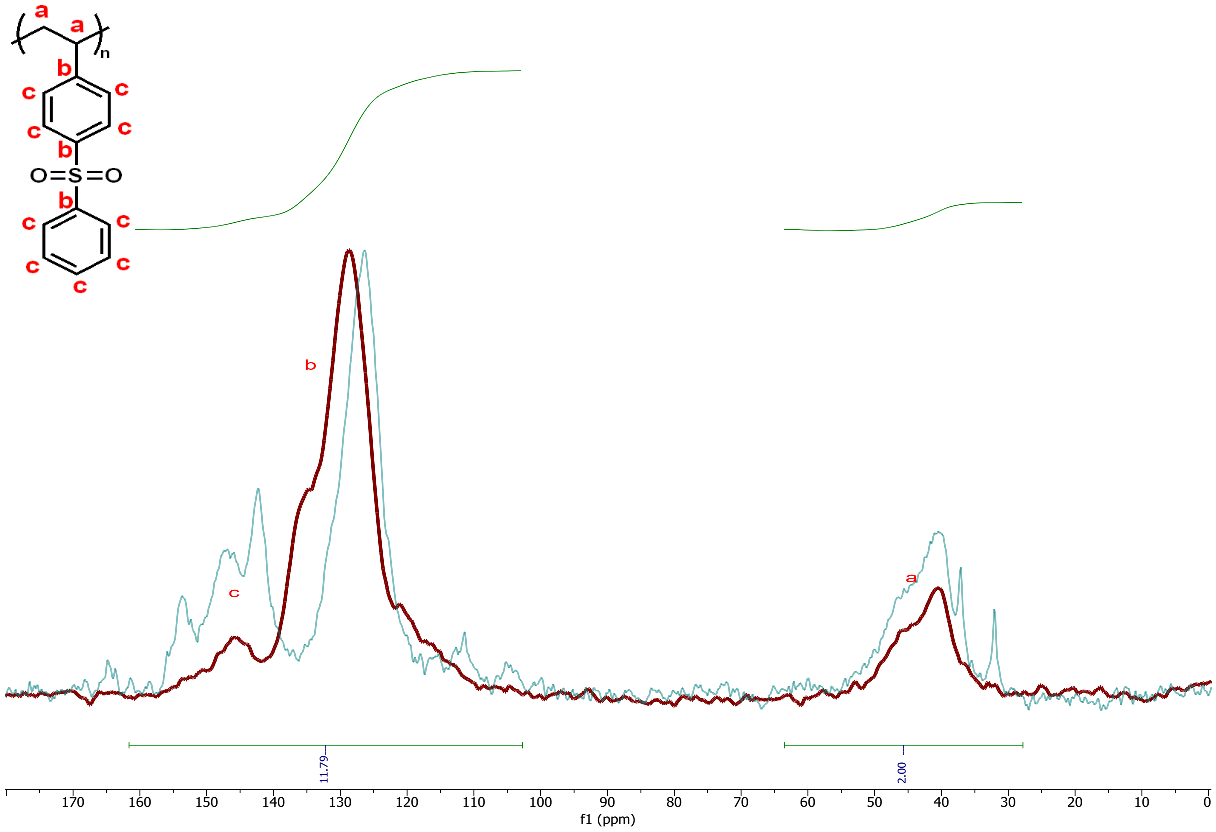

Figure S10. Direct-polarization solid-state ^{13}C NMR spectrum of the polystyrene phenyl sulfone. The red and green spectra represent purified and crude samples, respectively.

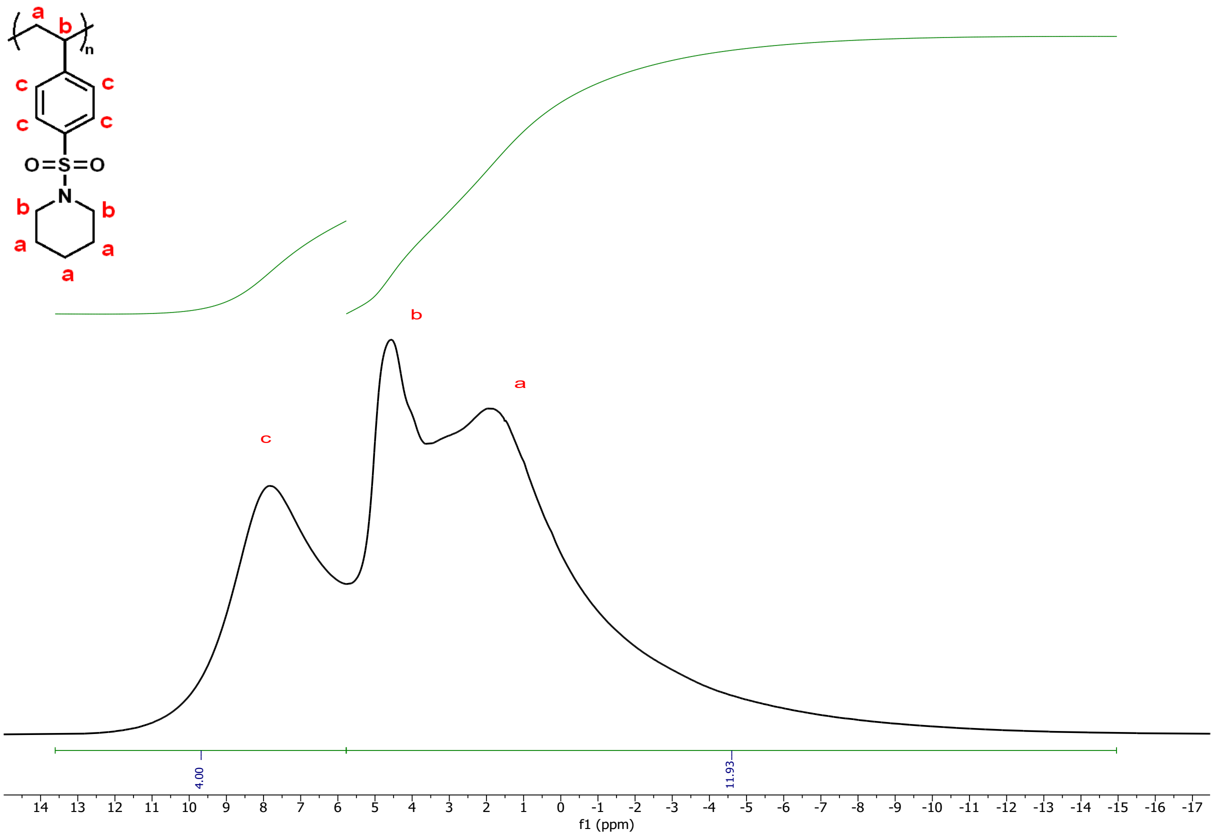

Figure S11. Solid-state ^{1}H NMR spectrum of the polystyrene sulfonyl piperidine.

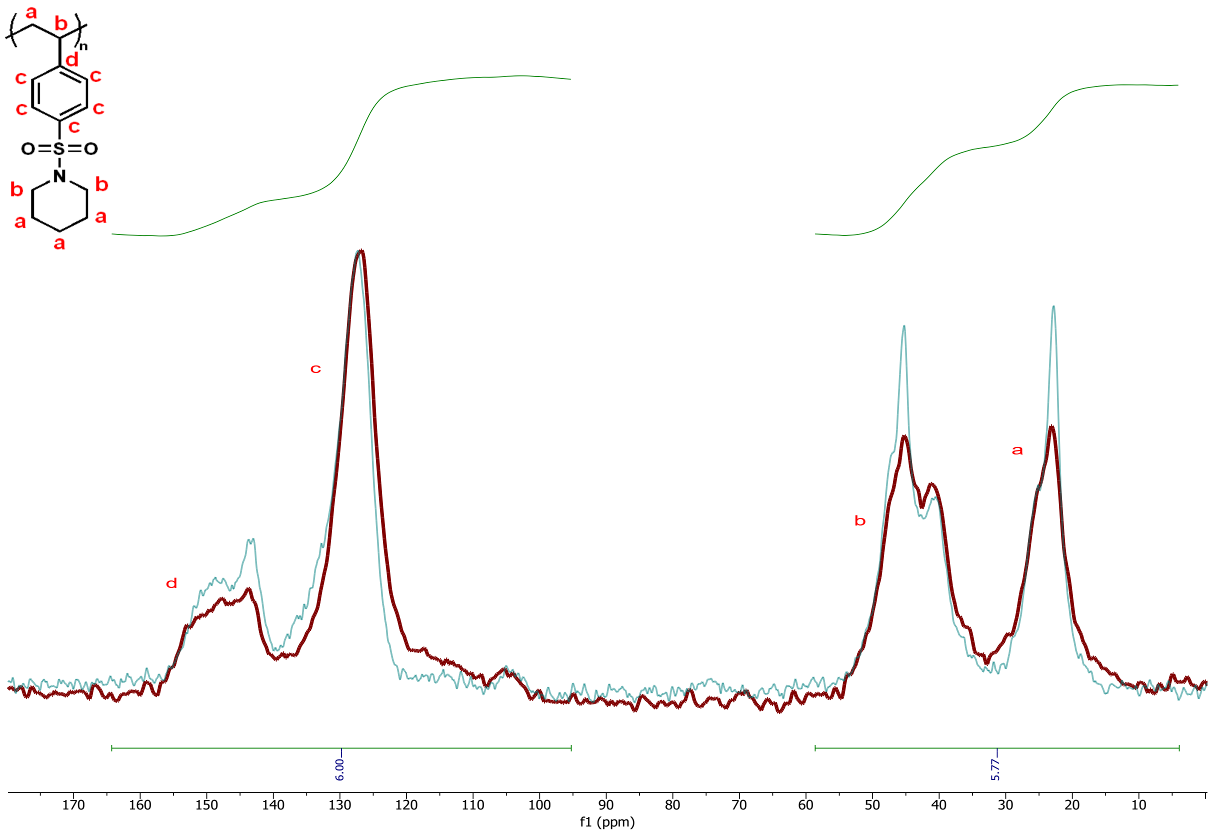

Figure S12. Direct-polarization solid-state ^{13}C NMR spectrum of the polystyrene sulfonyl piperidine. The red and green spectra represent purified and crude samples, respectively.

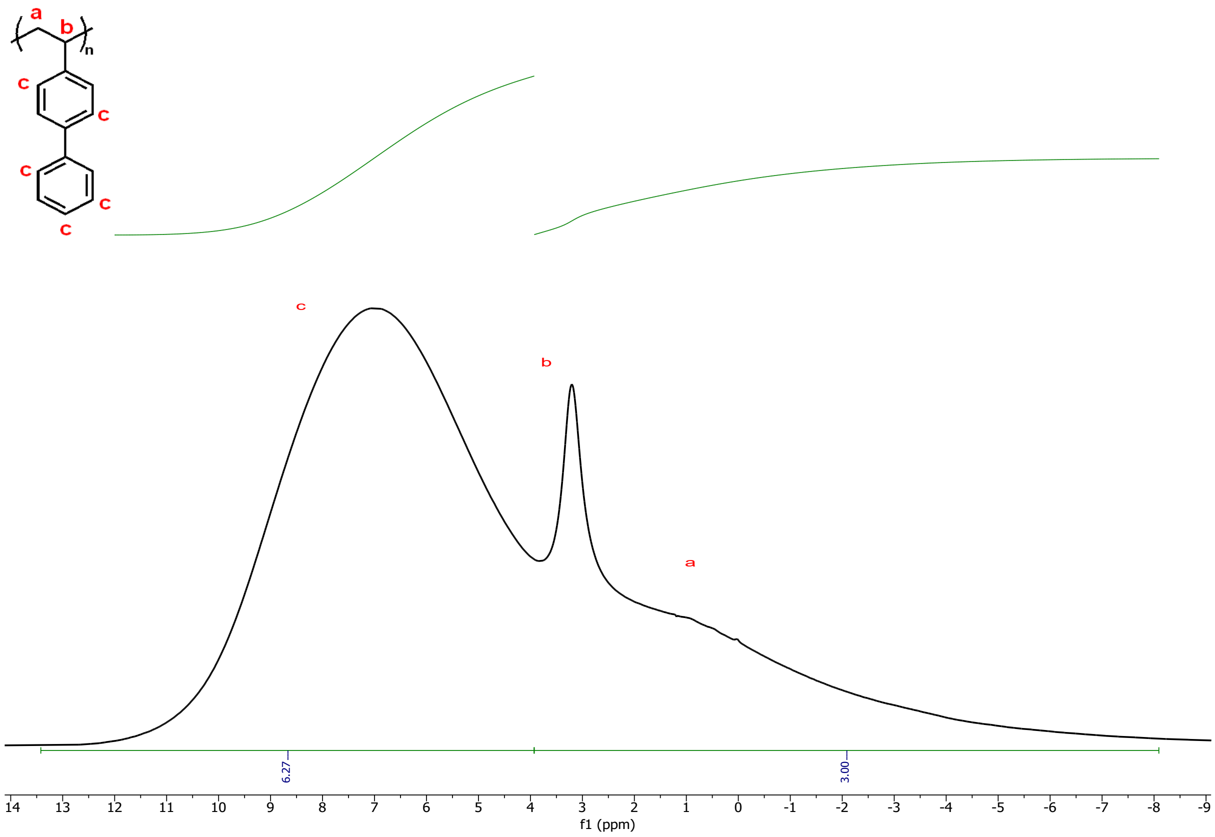

Figure S13. Solid-state ^{1}H NMR spectrum of the polyphenyl styrene.

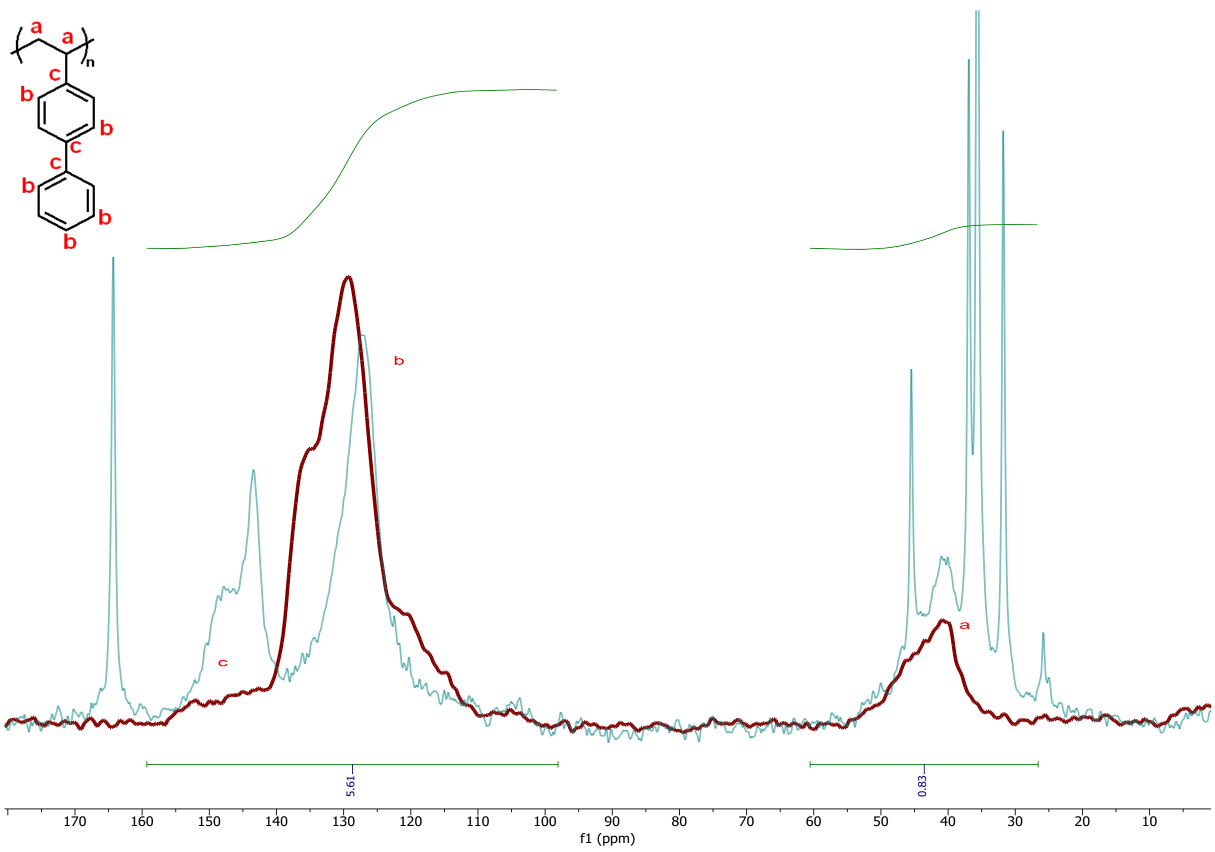

Figure S14. Direct-polarization solid-state ^{13}C NMR spectrum of the polyphenyl styrene. The red and green spectra represent purified and crude samples, respectively.

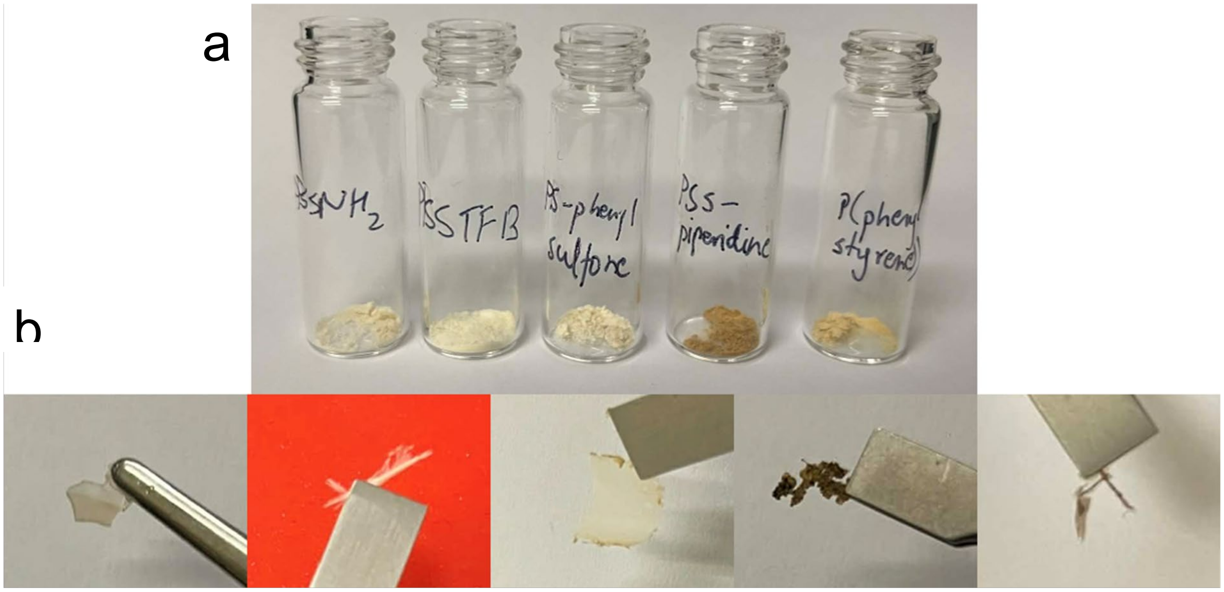

Figure S15. Processability of functionalized polymers. (a) Powder form of functionalized polymers (b) Hot-pressed functionalized polymers. From left to right: polystyrene sulfonamide, polystyrene trifluorobutyl sulfone, polystyrene phenyl sulfone, polystyrene sulfonyl piperidine, polyphenyl styrene.

References

(1) Torchia, D. A. The measurement of proton-enhanced carbon-13 T1 values by a method which suppresses artifacts. *Journal of Magnetic Resonance (1969)* **1978**, *30* (3), 613–616.

(2) Fung, B.; Khitrin, A.; Ermolaev, K. An improved broadband decoupling sequence for liquid crystals and solids. *Journal of magnetic resonance* **2000**, *142* (1), 97–101.
